# Cortical structural changes of morphometric similarity network in early-onset schizophrenia correlate with specific transcriptional expression patterns

**DOI:** 10.1186/s12916-023-03201-1

**Published:** 2023-12-05

**Authors:** Guanqun Yao, Ting Zou, Jing Luo, Shuang Hu, Langxiong Yang, Jing Li, Xinrong Li, Yuqi Zhang, Kun Feng, Yong Xu, Pozi Liu

**Affiliations:** 1 School of Medicine, Tsinghua University, Beijing, 100084 China; 2 Department of Psychiatry, Tsinghua University Yuquan Hospital, Shijingshan District, 5 Shijingshan Road, Beijing, China; 3 School of Life Sciences, University of Electronic Science and Technology of China, Chengdu, 611731 China; 4 Shanghai Mental Health Center, Shanghai, 200030 China; 5 College of Humanities and Social Science, Shanxi Medical University, Taiyuan, 030001 China; 6 School of Mental Health, Shanxi Medical University, Taiyuan, 030001 China; 7 Department of Psychiatry, the First Hospital of Shanxi Medical University, Taiyuan, 030001 China; 8 Department of Mental Health, Shanxi Medical University, Taiyuan Central Hospital of Shanxi Medical University, 256 Fen Dongnan Road, Xiaodian District, Taiyuan City, Shanxi Province China

**Keywords:** Early-onset schizophrenia, Morphometric similarity network, Neuroanatomical subtypes, Transcriptional signatures, Cell type-specific signatures

## Abstract

**Background:**

This study aimed to investigate the neuroanatomical subtypes among early-onset schizophrenia (EOS) patients by exploring the association between structural alterations and molecular mechanisms using a combined analysis of morphometric similarity network (MSN) changes and specific transcriptional expression patterns.

**Methods:**

We recruited 206 subjects aged 7 to 17 years, including 100 EOS patients and 106 healthy controls (HC). Heterogeneity through discriminant analysis (HYDRA) was used to identify the EOS subtypes within the MSN strength. The differences in morphometric similarity between each EOS subtype and HC were compared. Furthermore, we examined the link between morphometric changes and brain-wide gene expression in different EOS subtypes using partial least squares regression (PLS) weight mapping, evaluated genetic commonalities with psychiatric disorders, identified functional enrichments of PLS-weighted genes, and assessed cellular transcriptional signatures.

**Results:**

Two distinct MSN-based EOS subtypes were identified, each exhibiting different abnormal MSN strength and cognitive functions compared to HC. The PLS1 score mapping demonstrated anterior–posterior gradients of gene expression in EOS1, whereas inverse distributions were observed in EOS2 cohorts. Genetic commonalities were identified in autistic disorder and adult schizophrenia with EOS1 and inflammatory bowel diseases with EOS2 cohorts. The EOS1 PLS1- genes (Z < -5) were significantly enriched in synaptic signaling-related functions, whereas EOS2 demonstrated enrichments in virtual infection-related pathways. Furthermore, the majority of observed associations with EOS1-specific MSN strength differences contributed to specific transcriptional changes in astrocytes and neurons.

**Conclusions:**

The findings of this study provide a comprehensive analysis of neuroanatomical subtypes in EOS, shedding light on the intricate relationships between macrostructural and molecular aspects of the EOS disease.

**Supplementary Information:**

The online version contains supplementary material available at 10.1186/s12916-023-03201-1.

## Background

Schizophrenia (SCZ) is a severely progressive psychiatric disease, characterized by both positive and negative symptoms. It is known to affect approximately 1% to 3% of the population worldwide [1]. The diagnosis of SCZ is heterogeneous and therefore it is highly subjective and inaccurate to classify its diversity based only on clinical symptoms [2]. The objective definition of biological subtypes using neuroanatomical data is crucial for further progress on the disease. Of note, early-onset SCZ (EOS), a rare form of SCZ with the onset of the disease before the age of 18 years, exhibits a poorer prognosis [3]. To date, the specific architectural brain changes and the biological pathogenesis of EOS have remained unclear [4].

To date, numerous studies have focused on the application of magnetic resonance imaging (MRI) for detecting specific architectural changes in SCZ patients. Multiple brain abnormalities, including structural abnormalities in the cortical areas, slow-growing white matter, and disrupted functional connections [5, 6], have been linked to altered social cognitions and executive functions, as well as sensorimotor processing in SCZ [7, 8]. MRI studies of EOS revealed that EOS patients exhibited abnormal structural and functional integration [9] and abnormal development of the ventral occipitotemporal gray matter network associated with the social perception system [10]. This finding supports the hypotheses of abnormal neural development and dysconnectivity in EOS, but the specific abnormal pattern of brain connectivity and corresponding biological interpretation in EOS are absent.

Data-driven modeling of the objective neuroanatomical data showed two distinct but stable subtypes of brain atrophy in adult-SCZ that exhibited different clinical characteristics and therapeutic effects [11]. Similarly, another study identified two distinct neuroanatomical subtypes of adult-SCZ based on volume measurements of gray matter, white matter, and cerebrospinal fluid, characterized by a broad volume reduction and larger basal ganglia and internal capsule [12]. However, the majority of the studies involved only single MRI morphometric and anatomical features, such as cortical thickness, volume, and curvature. Multiple MRI parameters could be utilized to obtain a better classification. Morphometric similarity mapping (MSN), a classical analytic procedure, quantifies the similarity across multiple cortical areas by combining multiple MRI parameters to construct whole-brain morphometric networks for each subject [13, 14]. MSN has been widely used to detect macroscale cortical structural abnormalities in several mental disorders. Additionally, heterogeneity through discriminant analysis (HYDRA), a novel machine learning approach, is used to cluster disease effects by modeling differences with healthy controls (HC) instead of directly clustering the patients [15]. A combination of MSN with HYDRA is a promising technique and explores the neuroanatomical subtypes of EOS to deepen our understanding of the early pathophysiology of SCZ.

The neurodevelopmental theory of SCZ posited that environmental and genetic risk factors, ranging from prenatal periods to adolescent stages, remarkably influenced the typical developmental trajectory of brain tissues, leading to psychotic symptoms during adolescence or early adulthood [16]. Furthermore, genetic factors are known to impact mental diseases by shaping brain connectivity through connectomes [17], and corresponding brain-wide transcriptional expression atlases can serve as the bridge connecting the brain connectomes with biological functions [18]. Furthermore, studies combining the MSN analysis and gene transcripts have revealed the potential relationships between macroscale architectural abnormalities and specific transcriptional expression patterns in different mental disorders, such as major depressive disorder (MDD) [19], adult-SCZ [20], and autism [21]. However, further studies are warranted to understand the combined evaluation of MSN differences and regional gene expression patterns for EOS, explore the potential pathogenesis, and develop novel therapeutic targets for individuals with EOS.

We studied the connection between molecular mechanisms and structural alterations in different EOS subtypes by associating EOS-related MSN abnormalities with transcriptional data. First, the HYDRA method was used to identify two distinct EOS subtypes based on MSN characteristics. Different abnormal MSN patterns between EOS subtypes and HC were revealed. For each subtype, we investigated the relationship between EOS-related regional changes in the MSN and corresponding gene expression patterns using the Allen Human Brain Atlas (AHBA) and in *situ* hybridization (ISH) data to identify EOS-related genes. Subsequently, we performed Spearman’s correlation analysis between published differential gene expression (DGEs) patterns of other mental disorders and EOS-related MSN differences. Finally, functional enrichment analysis was applied to decipher the ontological pathways of EOS-related genes. Specific cell types were mapped to estimate their contribution to the transcriptomic relationship with EOS-related MSN changes in different EOS subtypes. In conclusion, our findings generated an in-depth understanding of EOS subtypes, revealing a complex connection between MSN macrostructural changes and specific transcriptional expression patterns.

## Methods

### Participants

A total of 206 participants aged 7 to 17 years were recruited in the study. Of these, 100 patients diagnosed with EOS were assessed by two psychiatrists from the First Hospital of Shanxi Medical University, whereas 106 HC were recruited from nearby communities. The diagnosis of EOS was based on the Diagnostic and Statistical Manual of Mental Disorders Structured Clinical Interview, Fourth Edition (DSM-IV) and was confirmed by two psychiatrists through a structured clinical interview after a follow-up of at least 6 months. Subsequently, each participant underwent an MRI. Among the 100 EOS patients, 21 were missing information on the Positive and Negative Symptom Scale (PANSS), a standardized scale designed to evaluate the severity of SCZ [22]. Furthermore, 56 subjects (21 HC and 35 EOS patients) were evaluated using the Wechsler Intelligence Scale for children-Chinese Revised (WISC-CR), which is widely used in Chinese hospitals to assess the intellectual development of minors aged 6 to 16 years [23]. The inclusion criteria for EOS were as follows: (i) age from 7 to 17 years, right-handed, and of Han nationality, (ii) meeting the DSM-IV diagnostic standards for SCZ, and (iii) initial diagnosis of SCZ without any previous use of psychotropic medication. The exclusion criteria were as follows: (i) the presence of comorbid axis-I or axis-II disorders, (ii) duration of SCZ exceeding 1 year, (iii) severe organic brain or systemic diseases, and (iv) history of claustrophobia or the presence of metal implants. In addition, HC were recruited with no mental illness or family-inherited history.

### Imaging acquisition

Imaging data were collected from the First Hospital of Shanxi Medical University using a 3.0 Tesla MRI scanner (MAGNETOM Verio 3 T, Siemens Medical Solutions, Germany). We used a three-dimensional (3D) fast spoiled gradient-echo (FSPGR) sequence to acquire high spatial resolution T1-weighted anatomical images by setting the “matrix” as 240 × 256, “field of view” as 225 × 240 mm^2^, voxel size = 0.9 × 0.9 × 1.2 mm^3^, “slice thickness” as 1.2 mm (no gap), “time echo (TE)” as 2.95 ms, “time repetition (TR)” as 2,300 ms, “flip angle” as 9 degrees, and 160-axial slices.

### Imaging preprocessing

The T1-weighted (T1w) images in surface-based space were preprocessed using FreeSurfer (v6.0, http://surfer.nmr.mgh.harvard.edu/) [24]. In brief, preprocessing involved skull stripping, tissue segmentation, partitioning of hemibrains and subcortical structures, and creation of gray white interfaces and pial surfaces. Participants with poor-quality scans were excluded from the study. Furthermore, the Euler number and total intracranial volume (TIV) were computed for each T1w image [25].

### Construction of MSN

In total, 308 spatially adjacent areas were obtained by segmenting 68 cortical areas in the D-K atlas [13]. This parcellated D-K atlas was transformed to each participant’s surface. For each region, five features were derived from T1w imaging, including surface area, cortical thickness, gray matter volume, Gaussian curvature, and mean curvature [20]. Each morphometric feature vector underwent *z*-normalization across the areas to accommodate variations in value distributions among features. Subsequently, Pearson’s correlation analysis was conducted on morphometric feature vectors to establish pairwise correlations between cortical regions, generating a 308 × 308 MSN for each participant without applying any thresholding. The average weighted correlation coefficient between a special area and all other areas was calculated to evaluate the MSN strength and quantify the connectivity strength of that region.

### Subtyping EOS with HYDRA

The HYDRA was used to identify subtypes within the MSN strength [15]. HYDRA differentiates the subtypes within an EOS population by comparing the EOS patients and HC. Unlike conventional supervised learning methods, such as support vector machines and random forests, which cannot differentiate between different subtypes of EOS patients, HYDRA can simultaneously classify and cluster the subtypes. Classification involves segregating HC from EOS patients using the linear maximum-margin classifiers to define a polytope. Subtyping involves clustering the EOS patients based on their association with different faces of a polyhedron called a hyperplane. HYDRA analyses were conducted using the following parameters: 50 iterations alternating between hyperplane estimation and cluster estimation, 20 consensus steps for clustering, a regularization parameter of 0.25, and tenfold cross-validation. Furthermore, the similarity between clustering results was assessed using the Adjusted Rand Index (ARI), accounting for clustering stability across the tenfold cross-validation [12]. The ARI corrects for chance grouping, providing a more conservative assessment of overlap (Additional File 1: HYDRA method) [26]. We validated the stability and homogeneity of subtypes in adult-SCZ using HYDRA and MRI image datasets, consisting of 185 adult-SCZ and 227 HC, from Morgan et al.’s study [20]. These datasets were applied to reidentify disease subtypes and compare the difference of MSN strength with adult-SCZ subtypes.

### Case–control analysis of MSN strength for EOS subtypes

We next used a linear regression model (LRM) using the MSN strength as the dependent variable and incorporating age, sex, and TIV as covariates to assess the EOS subtypes and HC differences. The global MSN strength, calculated as the average MSN strength across all regions, was used for each participant. To compare the MSN strength in each region (MSN_i_) between EOS subtypes and HC, the following formula was utilized: MSN_i_ = intercept + β1 × age + β2 × sex + β3 × TIV. Next, two-sided *t*-tests (contrast = subtyping EOS-HC) were performed. The Bonferroni correction (*p* < 0.05) was applied to account for multiple comparisons.

### Acquisition and preprocessing of regional gene expressional profiles

Transcriptional profiles of brain tissues, including gene expression data from six postmortem brains at 3,702 spatially distinct locations were obtained from the AHBA dataset (http://human.brain-map.org) [27]. The dataset comprised 58,692 probes according to 20,737 genes and provided detailed annotations of spatial locations in the brain to match transcriptional profiles with specific brain regions. Subsequently, we preprocessed the AHBA dataset using the Abagen toolbox (https://github.com/rmarkello/abagen) following the standard protocols [28]. The preprocessing workflow involved (i) converting the microarray probes into gene symbols, (ii) excluding low-intensity probes with expression below the background noise in over 50% of samples, (iii) selecting probes with the highest homogeneities of regional variation for genes targeted by multiple probes, (iv) assigning the samples to brain regions within 2 mm Euclidean distance from the region boundary, and [5] normalizing gene expression across tissue samples using a scaled robust sigmoid function. We focused our analysis on the left hemisphere dataset due to the limited availability of right hemisphere data in the AHBA dataset [29]. Consequently, we obtained a transcriptional matrix consisting of 152 regions and 15,631 gene expression levels.

### Potential connection of regional changes in the transcriptome and MSN strength

The strong co-expression phenomenon among genes and the influence of spatial distance on gene expression (i.e., closer regions exhibiting more similar expressional models) suggested that the dimension of regional gene expressional profiles can be effectively reduced to several special principal components accounting for the majority of expression variability. Hence, we applied partial least squares (PLS) regression to explore the spatial relationships among the expression of all 15,631 genes (predictor variables) and *t-*value maps from 152 cortical regions (response variables) [30]. The first PLS component (PLS1) was identified as a linear combination of gene expression exhibiting the strongest association with *t*-statistic maps. We performed a permutation test with 10,000 iterations to assess whether the covariance between transcriptomic scores and *t*-statistic maps in the PLS1 component exceeded chance expectations. Moreover, we used bootstrapping (with 10,000 bootstrap samples) to evaluate the variability of each gene in the PLS1 component [19]. The *Z* values were calculated as the ratio of each region’s expression weight to its bootstrap standard error, and all genes were ranked based on their weights to PLS1 [20]. Significant genes were divided into two extremely different lists, called PLS1 + genes (*Z* > 5) and PLS1- genes (*Z* < -5) depending on their PLS1 weight values. The correlation analysis between PLS1 scores and *t*-statistic maps was performed by Spearman’s correlation method and evaluated by spatial correlation [31].

We further evaluated the weight of MSN in EOS subtypes by performing spatial correlation analysis between *t*-statistic maps and SCZ-related genes in ISH levels. The SCZ-related genes were defined from the “In Situ Hybridization in the Human Brain Atlas” in the AHBA database (help.brain-map.org/display/humanbrain/Documentation), integrating SCZ-related available datasets based on the published literature [19]. A total of 77 unique SCZ-related genes were selected from two datasets: “The 1,000 Gene Survey in Cortex” (*n* = 26) and “The Schizophrenia Study” (*n* = 67). Afterword, we acquired the overlapping genes between 77 SCZ-related and 15,631 background genes. Spatial correlation analysis was further estimated between the expression of overlapping genes and case–control *t*-values of MSN in the left hemisphere. Statistical significance was set as a bilateral *p*-value < 0.05, corrected using the false discovery rate (FDR) for multiple comparisons.

### Correlation analysis of PLS1 genes and other brain disorders

We next performed the correlation analysis between PLS1-weight values and DGE values of dysregulated genes of other brain-disorder diseases to explore whether transcriptionally dysregulated genes in other brain disorders were consistent with abnormal cortical regions that are morphometrically related to EOS. We obtained a list of dysregulated genes from six brain disorders according to Gandal et al. [32], including alcohol abuse disorder (AAD), major depressive disorder (MDD), adult-SCZ, bipolar disorder (BD), autism spectrum disorder (ASD), and inflammatory bowel disease (IBD). The corresponding and overlapping dysregulated genes were acquired, and the log-2 (fold change) values were used to conduct the correlation analysis with permutation tests. We next performed the MAGMA analysis for gene set enrichment for similar neuropsychiatric disorders to evaluate functional enrichments of these psychiatric disorders at genetic levels. Genome-wide association study (GWAS) summary datasets were obtained from the FinnGen database (Round 9, https://www.finngen.fi/en/access_results), and the enrichment analysis was conducted using the MAGMA software (https://github.com/SarahMorgan/Morphometric_Similarity_SZ/blob/master/magma_enrichments.md) (Additional file 2: Table S1).

### Functional enrichment evaluation of PLS1 positive or negative genes

The PLS1 genes (*Z* > 5 or *Z* < -5) were applied to conduct the functional enrichment analysis, including the evaluation of biological processes (BP) of gene ontology (GO) and the enrichment of Kyoto Encyclopedia of Genes and Genomes (KEGG) pathways. The GO analysis was performed using the online software: g:Profiler (https://biit.cs.ut.ee/gprofiler/gost) [33], and KEGG pathway enrichment was achieved using the ClueGO plug-in of the Cytoscape software [34]. The BPs and pathway networks were visualized using the “ggplot2” R package and Cytoscape software.

### Investigating regional cellular changes by mapping subtyping EOS-related genes into cell types

We overlapped the PLS1 genes with the list of genes of different cortical cells to map cellular heterogenicity onto brain regional changes in MSN. We collected the data from five independent single-cell studies conducted on human postmortem cortical tissues to obtain the list of genes for seven canonical cell types, including endothelial cells, astrocytes, microglia, oligodendrocytes, oligodendrocyte precursors (OPCs), and excitatory and inhibitory neurons [14]. We further excluded gene sets of three studies without subdivision to neurons (two studies) and including the annotation of pericytes (one study). This approach eliminated any potential biases arising from the differences in data acquisition methods, thresholding identification, or analysis techniques. Finally, we used the single sample gene set enrichment analysis (ssGSEA) algorithm to obtain cellular quantificational levels (ssGSEA scores) in each brain region based on the above gene sets of special cells, with the “gsva” function and “Gaussian” methods using the GSVA R package [35].

Next, we overlapped the PLS- or PLS + rank gene lists and gene sets of the above cell types to subtype EOS-related genes identified by PLS regression into specific brain cells. We performed permutation tests to acquire the *p-*value for the number of overlapping genes in each cell type, followed by the application of FDR correction with *p* < 0.05. The enrichment analysis of overlapping genes was conducted using Metascape (https://metascape.org), an online tool enabling automated meta-analysis to acquire enriched biological processes and pathways based on 40 independent knowledge bases [36]. All significant GO terms or KEGG pathways of each cell were identified by FDR correction with *p* < 0.05. Moreover, we further evaluated developmental time windows across different brain regions by conducting a developmental gene expression enrichment analysis using the cell-type specific expression analysis (CSEA) tool (http://genetics.wustl.edu/jdlab/csea-tool-2/) [37].

## Results

### Demographic characteristics and two EOS subtypes

**Fig. 1:**
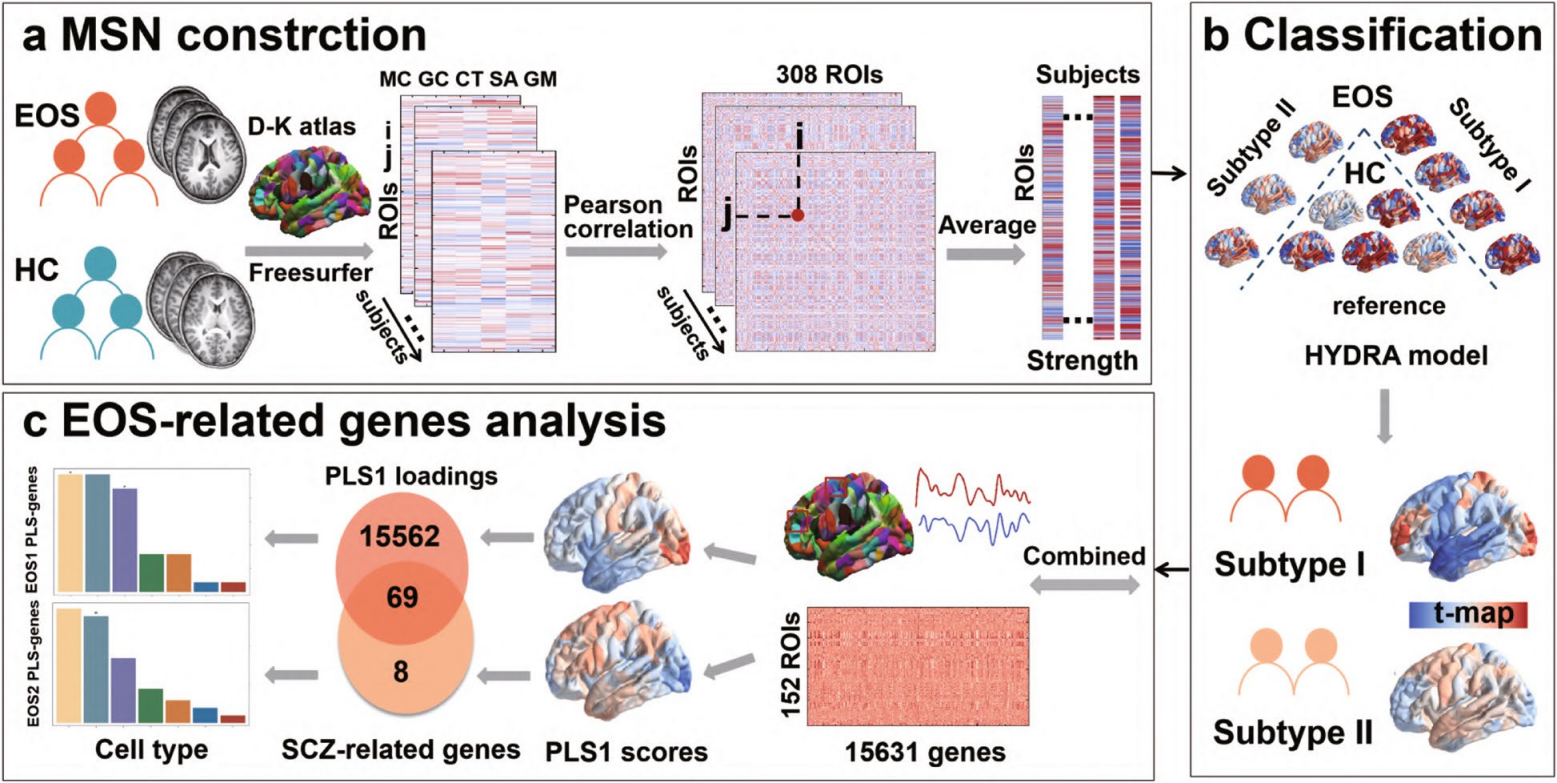
The workflow of this study. **a**. MSN construction. The 308 × 308 matrix for MSN was calculated across multiple macrostructural features (surface area, cortical thickness, gray matter volume, Gaussian curvature, and mean curvature). The MSN strength was obtained by the average weighted correlation coefficients between a given region and all other regions. **b**. Classification. The HYDRA method was used to identify EOS subtypes within the MSN strength. **c**. EOS-related gene analysis. PLS regression was then used to identify imaging transcriptomic associations. The link between brain-wide gene expression and morphometric changes in different EOS subtypes was evaluated through PLS weight mapping, genetic commonalities evaluation with psychiatric disorders, functional enrichment of PLS weighted genes, and cellular transcriptional signature assessment

We used a combination of multiple cortical features and transcriptional data to establish the connections between gene expression and alterations in the MSN of EOS subtypes compared to HC (Fig. 1). The demographic analysis demonstrated no statistically significant differences in sex, age, TIV, or Euler number between EOS patients (mean age: 14.60 ± 1.95) and HC (mean age: 14.58 ± 2.46) (Table 1). The consistency of clustering assignments across different resolutions (2–10 clusters) was assessed using the ARI, which is known for its insensitivity to the number of clusters (K) [12]. The highest reproducibility was observed with a two-cluster solution (K = 2), yielding an ARI value of 0.62 (Additional file 1: Fig. S1). At resolutions of K = 3 to 10, the ARIs were lower than those at K = 2. Consequently, 53 EOS patients were assigned to EOS1, and 47 were assigned to EOS2. Based on these convergent findings, a subsequent analysis focused on the two EOS subtypes (EOS1 and EOS2). Furthermore, both EOS subtypes exhibited decreased verbal (VIQ), performance (PIQ), and full-scale intelligence quotient (FSIQ) scores compared with HC. The EOS1 displayed lower VIQ and FSIQ scores compared to EOS2 (Additional file 1: Fig. S2). Additionally, EOS1 and EOS2 exhibited no significant difference in sex, age, TIV, and PANSS scores.

**Table 1:** Clinical and demographic characteristics

Variable	HC (*n* = 106)	EOS (*n* = 100)	*p*	EOS 1 (*n* = 53)	EOS 2 (*n* = 47)	*p*
Age (year)	14.58 ± 2.46	14.60 ± 1.95	0.426^a^	14.90 ± 1.66	14.27 ± 2.21	0.223^a^
Sex (M/F)	76/30	60/40	0.068^b^	21/32	19/28	0.935^b^
TIV	1445.00 ± 125.00	1490.00 ± 158.10	0.072^a^	1470.00 ± 149.50	1513.00 ± 166.00	0.187^a^
Euler number	-67.66 ± 29.61	-73.36 ± 44.60	0.721^a^	-73.51 ± 45.91	-73.19 ± 43.57	0.870^a^
PANSS total		60.91 ± 15.79		60.14 ± 14.20	61.83 ± 17.68	0.643^a^
PANSS positive		15.00 ± 5.10		14.70 ± 4.77	15.36 ± 5.51	0.653^a^
PANSS negative		14.09 ± 6.02		13.98 ± 6.05	14.22 ± 6.08	0.759^a^
PANSS general		31.82 ± 8.40		31.47 ± 7.50	32.25 ± 9.46	0.726^a^

*HC* Healthy controls, *EOS* Early-onset schizophrenia, *M/F* Male/female, *TIV* Total intracranial volume, *PANSS* Positive and Negative Symptom Scale

^a^Mann-Whitney *U*-test (two-sided)

^b^Chi-square test

### Subtyping EOS-related changes in MSN strength

**Fig. 2:**
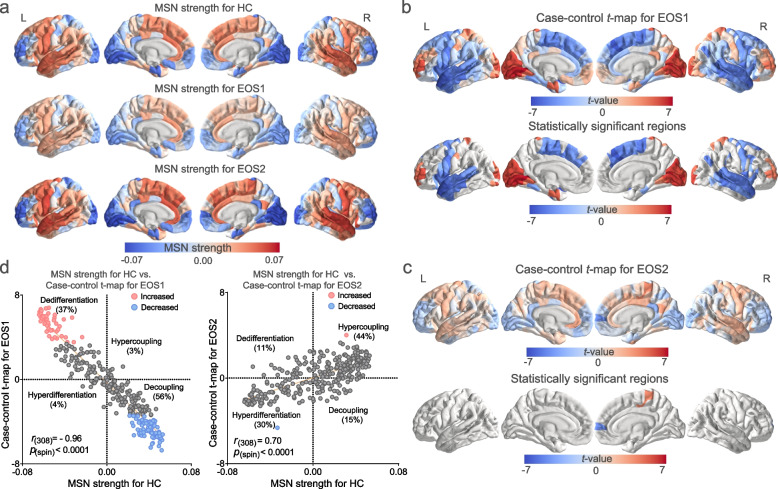
Subtyping EOS-related regional changes in MSN strength. **a**. The MSN strength of EOS subtypes and HC. **b-c**. Case–control comparison of MSN strength for EOS1 and EOS2. **d**. Scatterplot of the control MSN strength and case–control *t*-map

The temporal and frontal lobes displayed high MSN strength, whereas the somatosensory and occipital cortices exhibited low MSN strength (Fig. 2a). The global MSN strength of EOS1 was reduced compared with HC, whereas the global MSN strength of EOS2 did not differ significantly from HC (Additional file 1: Fig. S3). Specific cortical regions exhibited significant differences in the MSN strength between EOS1 and HC (Additional file 2: Table S2, Fig. 2b). Significantly decreased MSN strength in EOS1 was found in the temporal cortex, precentral gyrus, paracentral gyrus, superior frontal gyrus, and insula. Conversely, significantly increased MSN strength in EOS1 was observed in the lateral occipital gyrus, medial frontal gyrus, left entorhinal cortex, superior parietal gyrus, cuneus, postcentral gyrus, and lingual gyrus. Moreover, we found two cortical regions with significant MSN strength differences between EOS2 and HC (Additional file 2: Table S2, Fig. 2c). Significantly decreased MSN strength in the EOS2 subtype was observed in the right paracentral gyrus (part 3), whereas significantly increased MSN strength was observed in the right superior frontal gyrus (part 2) in the EOS2 subtype. We next explored the differences in MSN strength between EOS1 and EOS2 subtypes. Compared to the EOS2 subtype, the EOS1 subtype exhibited decreased MSN strength in the superior frontal gyrus, middle frontal gyrus, insula, and anterior central gyrus, and increased MSN strength in the lateral occipital cortex, lingual gyrus, and cuneus (Additional file 1: Fig. S4).

Abnormal MSN strength was observed in functional Yeo 7 networks [38] (Additional file 1: Fig. S5a) and von Economo atlas (Additional file 1: Fig. S5b) [39] between EOS subtypes and HC. For the Yeo 7 functional networks, the EOS1 subtype displayed significantly increased MSN strength in the visual network and decreased MSN strength in the somatomotor, ventral attention, and default mode networks, whereas the EOS2 subtype displayed significantly increased MSN strength in the somatomotor network. Concerning the von Economo atlas, EOS1 exhibited significantly increased MSN strength in the secondary and primary sensory networks and decreased MSN strength in the primary motor, association, and insular networks. whereas EOS2 displayed significantly increased MSN strength in the primary motor network.

We used a quadratic non-linear model to construct the global MSN strength and the MSN strength of the functional network to explore the developmental trajectories of EOS subtypes from childhood to adolescence. The reason for selecting this approach is because age exerts non-linear patterns on the brain [40]. Thus, the default network of HC demonstrated a trend of an initial increase followed by a gradual decline from childhood to adolescence. The limbic and default network of EOS1 exhibited a rapid increase, followed by a gradual decline. Furthermore, the global strength and ventral attention network of EOS2 displayed a pronounced and consistent decline (Additional file 1: Fig. S6).

The MSN strength of HC and the case–control *t*-map of EOS1 exhibited a negative and spatial correlation (*r*_(308)_ = -0.96, *p*_spin_ < 0.0001) (Fig. 2d), suggesting that more connected regions exhibited larger case–control differences [14]. In addition, 56% of the positive MSN strength in HC and the negative *t*-values in the EOS1 were in decoupling, and 37% of the negative MSN strength in HC and the positive *t*-values in the EOS1 displayed dedifferentiation. However, EOS2 displayed the opposite pattern. The MSN strength of HC and the case–control *t*-map of EOS2 were positively and spatially correlated (*r*_(308)_ = 0.70, *p*_spin_ < 0.0001) (Fig. 2d). We found that 30% of negative MSN strength in HC and negative *t*-values in the EOS2 exhibited hyperdifferentiation, and 44% of positive MSN strength in HC and positive *t*-values in the EOS2 displayed hypercoupling. Spearman’s correlation analysis, conducted to assess the association between abnormal MSN strength and symptoms, revealed no significant correlations following Bonferroni’s correction (Additional file 2: Table S3). Additionally, we explored the spatial correlation between case–control *t*-maps of EOS subtypes and correlation coefficient maps of MSN strength and PANSS. In EOS1 patients, the case–control *t*-map displayed a significant spatial and positive correlation with statistical maps of MSN strength and PANSS positive and total scores but not with negative scores. In EOS2 patients, the case–control *t*-map exhibited significant spatial and negative correlation with statistical maps in PANSS positive and total scores, whereas positive correlation with statistical maps in PANSS negative scores (Additional file 1: Fig. S7).

In addition, we investigated the spatial correlation of case–control *t*-maps of the MSN strength between EOS subtypes and adult-SCZ in Morgan et al.’s study. EOS1 exhibited a significant spatial and positive correlation, whereas EOS2 displayed a significant spatial and negative correlation with case–control *t*-map of adult-SCZ (Additional file 1: Fig. S8). The positive/negative correlation suggested that EOS1 could be in a “classical SCZ” state, whereas EOS2 could fall in the “non-classical SCZ” state. The highest reproducibility of adult-SCZ was observed with a two-cluster solution (K = 2), yielding an ARI value of 0.52 (Additional file 1: Fig. S1) for subtype classification. The spatial correlation analysis revealed significant spatial and positive correlations of case–control *t*-map in both EOS1/2 and corresponding SCZ1/2 subtypes (Additional file 1: Fig. S9a-c). The positive correlations suggested that adult-SCZ could manifest two different subtypes similar to the EOS states. Moreover, type-I diseases displayed shared abnormal regions, encompassing increased regions in the superior parietal gyrus, lingual gyrus, cuneus, and postcentral gyrus, and reduced regions in the temporal gyrus, superior frontal gyrus, precentral gyrus, and insula (Additional file 1: Fig. S9d). However, no common abnormal regions were observed in type-II diseases.

### Transcriptional patterns related to regional changes in MSN strength

**Fig. 3:**
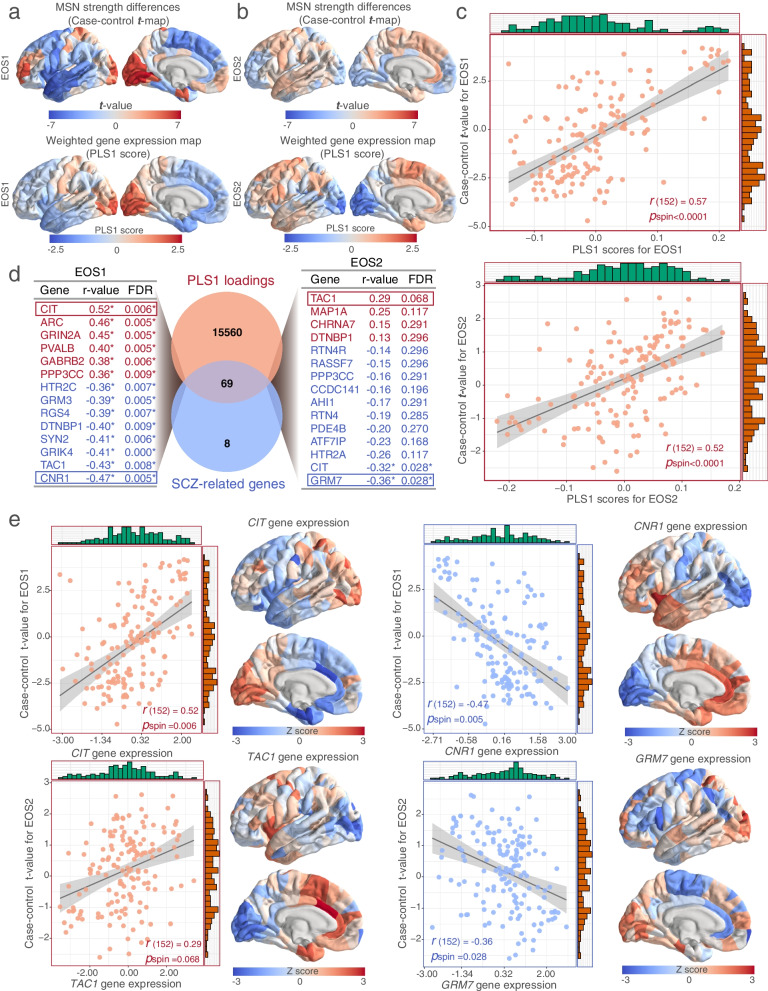
Transcriptional expression patterns related to differences in MSN strength. **a**. The distribution of differences in MSN strength and PLS1 scores in the left hemisphere of the EOS1 subtype. **b**. The distribution of differences in MSN strength and PLS1 scores in the left hemisphere of the EOS2 subtype. **c.** Scatterplots showing the significant spatial correlation between PLS1 scores and the case–control *t*-value maps of MSN strength in both EOS subtypes; EOS1, Spearman’s *r* = 0.57, *p*_spin_ < 0.0001; EOS2, Spearman’s *r* = 0.52, *p*_spin_ < 0.0001. **d-e**. The expression of SCZ-related genes from ISH datasets was positively or negatively associated with regional changes in MSN, including 6 positive genes (i.e., CIT, ARC, GRIN2A, PVALB, GABRB2, and PPP3CC) and 8 negative genes (i.e., HTR2C, GRM3, RGS4, DTNBP1, SYN2, GRIK4, TAC1, and CNR1). All r values were determined by Spearman’s correlation analysis, and *p* values were obtained from spatial correlation tests and adjusted with FDR correction

PLS regression was performed to uncover the gene expression patterns based on the distinct anatomical distributions of case–control *t*-maps in MSN strength. Therefore, the PLS1 of two subtypes effectively explained 39% and 25% of the variations in the macrostructural differences for EOS1 and EOS2 patients, respectively (*p*_perm_ < 0.0001) (Additional file 1: Fig. S10). The distribution of the PLS1 score-weighted map demonstrated an anterior–posterior gradient of gene expression in EOS1, whereas an inverse distribution in EOS2 cohorts was performed (Fig. 3a-b, Additional file 2: Table S4). This gradient reflected variations in the transcriptional architecture of the brain cortex and distinct expressional differences in EOS subtypes, which was also manifested in the EOS-related regional changes observed in the MSN strength map. Irrespective of EOS1 or EOS2 patients, the PLS1 scores exhibited a significant spatial correlation with the case–control *t*-value maps in MSN strength (EOS1, Spearman’s *r* = 0.57, *p*_spin_ < 0.0001; EOS2, Spearman’s *r* = 0.52, *p*_spin_ < 0.0001; Fig. 3c). Univariate one-sample *Z* tests were used to identified PLS1 weighted genes to represent transcriptional signatures for subsequent analysis, including 1,651 PLS1 + genes (Z > 5) and 1,743 PLS1- genes (Z < -5) for EOS1, and 79 PLS1 + and 318 PLS1- genes for EOS2 (all *p*_FDR_ < 0.0001) (Additional file 2: Table S5).

Next, we investigated the relationships between the published SCZ-related gene expression and corresponding regional changes in MSN strength. We acquired 77 SCZ-related genes by screening the keyword “schizophrenia” in the “Gene List” project from the ISH data in the AHBA database. In total, we screened 69 SCZ-related genes overlapping with 15,631 background genes and performed a spatial correlation analysis with case–control *t*-maps in MSN (Additional file 2: Table S6). In EOS1 cohorts, 14 SCZ-related genes exhibited *a* significant association (absolute(*r*) > 0.35, FDR correction, *p*_spin_ < 0.05) with homologous case–control *t*-values, including six positive genes (i.e., *CIT, ARC, GRIN2A, PVALB, GABRB2,* and *PPP3CC*) and eight negative genes (i.e., *HTR2C, GRM3, RGS4, DTNBP1, SYN2, GRIK4, TAC1*, and *CNR1*). However, only one SCZ-related gene (*TAC1*) exhibited a significant positive correlation in EOS2 cohorts, and two genes (*GRM7* and *CIT*) displayed a negative correlation with corresponding case–control *t*-maps (Fig. 3d). The expression of the top positively (or negatively) weighted genes was consistent with (or in contrast to) the distribution of variant regional changes in MSN strength, including *CIT* (Spearman’s *r* = 0.52, FDR correction, *p*_spin_ = 0.006), *CNR1* (Spearman’s *r* = -0.47, FDR correction *p*_spin_ = 0.005), *TAC1* (Spearman’s *r* = 0.29, FDR correction *p*_spin_ = 0.068), and *GRM7* (Spearman’s *r* = -0.36, FDR correction *p*_spin_ = 0.028) (Fig. 3e).

### Potential relationship between EOS-related changes in MSN strength and transcriptional dysregulation of other mental disorders

**Fig. 4:**
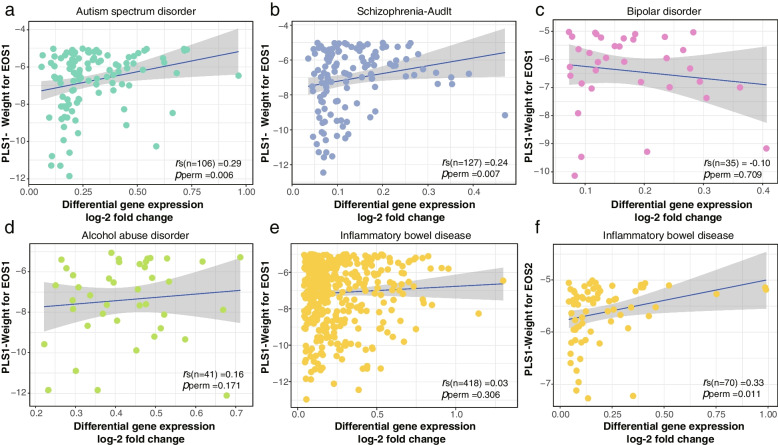
Correlation analysis between PLS1 weighted gene expressions of changes in MSN strength and transcriptional dysregulation of various mental disorders. **a-b**. In EOS1 patients, PLS1- weights exhibited significant positive associations with upregulated differential gene expression (DGE) in autism spectrum disorder (ASD) (*r*_s (106)_ = 0.29, adjusted *p*_perm_ = 0.006) and adult schizophrenia (*r*_s(127)_ = 0.24, adjusted *p*_perm_ = 0.007). **c-e**. There was no significant correlation with DGE in other mental disorders in EOS1 patients, including bipolar disorder (BD) (*r*_s(35)_ = -0.10, adjusted *p*_perm_ = 0.709), alcohol abuse disorder (AAD) (*r*_s(41)_ = 0.16, adjusted *p*_perm_ = 0.171) and inflammatory bowel disease (IBD) (*r*_s(418)_ = 0.03, adjusted *p*_perm_ = 0.306). **f**. In EOS2 patients, PLS1- weights exhibited significant positive associations with upregulated DGE in IBD (*r*_s(70)_ = 0.33, adjusted *p*_perm_ = 0.011). All *r* values were determined by Spearman’s correlation analysis, and *p* values were obtained from permutation tests and adjusted with FDR correction

To gain deeper insights into the potential relationship between regional changes and gene dysregulation in mental disorders, we performed the correlation analysis of PLS1- weighted genes and published disease-related DGEs (Additional file 2: Table S7). In EOS1 patients, we first obtained the DGEs of six mental disorders from Gandal’s study and further identified overlapping upregulated (log-2 [fold change] > 0) genes, including 2 for MDD, 106 for ASD, 127 for adult SCZ, 35 for BD, 41 for AAD, and 418 for IBD. The PLS1- gene weights displayed positive correlations with ASD-related and adult-SCZ-related DGE values, as confirmed by permutation tests: ASD (*r*_s (106)_ = 0.29, adjusted *p*_perm_ = 0.006) and adult-SCZ (*r*_s(127)_ = 0.24, adjusted *p*_perm_ = 0.007) (Fig. 4a-b). However, no significant correlation was found with other mental disorders, including BD (*r*_s(35)_ = -0.10, adjusted *p*_perm_ = 0.709), AAD (*r*_s(41)_ = 0.16, adjusted *p*_perm_ = 0.171) and IBD (*r*_s(418)_ = 0.03, adjusted *p*_perm_ = 0.306) (Fig. 4c-e). In contrast, in EOS2 cohorts, PLS1- gene weights were only positively related to IBD-related DGEs values (*r*_s(70)_ = 0.33, adjusted *p*_perm_ = 0.011), with few overlapping genes in the other five disorders (Fig. 4f, Additional file 1: Fig. S11). These results indicated that regional changes in two EOS subtypes exhibited distinct gene expression patterns of psychiatric disorders.

Moreover, the MAGMA gene-set enrichment analysis identified characteristic biological processes for these mental disorders, including energy metabolism-related processes (“cAMP catabolic process”, “electron transport”, “cytochrome c to oxygen”, and “thyroid hormone catabolic process”) in ASD, and pathways of amino-acid metabolism (“fatty acid homeostasis”, “aspartate metabolic process” and “aspartate biosynthetic process”) and neuronal signals (“cerebral cortex GABAergic interneuron fate commitment”) in adult-SCZ (Additional file 1: Fig. S12a-b). Additionally, the GWAS genes of BD were primarily enriched in neuronal interaction-related processes, including “regulation of dendrite development” and “beta-adrenergic receptor kinase activity”, whereas AAD was significantly enriched in Ion signal regulation related pathways, such as “nickel ion binding”, “localization within membrane”, and “regulation of potassium ion transport” (Additional file 1: Fig. S12c-d). In contrast, MDD was significantly associated with neuroreceptor signaling, including “ganglioside metabolic process” and “negative regulation of dopamine receptor signaling pathway”, whereas IBD was enriched in processes related to cellular development and immune activation, such as “endothelial cell development”, “JAK-STAT cascade involved in growth hormone signaling pathway” and “positive regulation of interleukin-1 beta production” (Additional file 1: Fig. S12e-f).

### Functional enrichment of genes correlated with regional changes in MSN strength

**Fig. 5:**
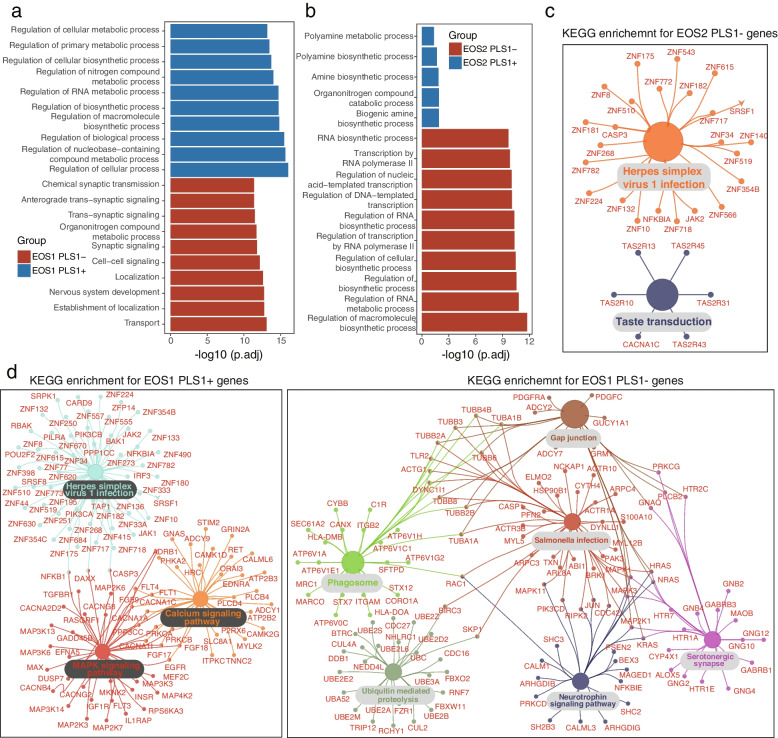
Functional enrichment of PLS1 weighted genes related to regional changes in MSN. **a-b**. Top 10 biological process (BP) terms of PLS1 + (Z > 5, *p*_FDR_ < 0.05) and PLS1- (Z <  − 5, *p*_FDR_ < 0.05) gene enrichment in the two EOS subtypes. **c-d**. KEGG pathway enrichment of PLS + and PLS- genes in the two EOS subtypes. The large circle nodes represent terms of the pathway, and small circle nodes represent related genes. The size of large nodes represents the -log10 (adjusted *p* values) of pathways, and lines indicate the relationships between genes and pathways

To further elucidate the functional characteristics of genes correlated with regional changes in MSN, we aligned GO and KEGG enrichment analyses with PLS1 weighted gene lists (Additional file 2: Table S8). For EOS1 patients, the column diagram displayed the top 10 significant BP terms, including “regulation of cellular metabolic process” and “regulation of RNA metabolic process” for PLS1 + genes and “synaptic signaling”, “cell–cell signaling”, “localization” and “nervous system development” for PLS- genes (Fig. 5a). In EOS2 cohorts, PLS1 + gene lists were only enriched in five BP, such as “polyamine metabolic process” and “polyamine biosynthetic process”, whereas PLS- genes exhibited similar enrichment in cellular and RNA metabolic processes as the PLS + genes in EOS1 patients (Fig. 5b). In addition, the KEGG analysis validated the enrichment of common pathways between EOS1 PLS1 + genes and EOS2 PLS1- genes, especially in the “Herpes simplex virus 1 infection” pathway, whereas no significant enrichment for PLS1 + genes were observed in EOS2 (Fig. 5c). The EOS1 PLS1 + genes were uniquely enriched in the “MAPK signaling pathway” and “calcium signaling pathway”, suggesting their involvement encephalic regional abnormities in EOS1 patients. In contrast, the EOS1 PLS1- genes were significantly enriched in synaptic signaling-related functions, such as serotonergic synapse, phagosome, gap junction, neurotrophin signaling pathway, and others (Fig. 5d).

### Transcriptional signature assessment for canonical brain cell types and specific developmental stages in EOS

**Fig. 6:**
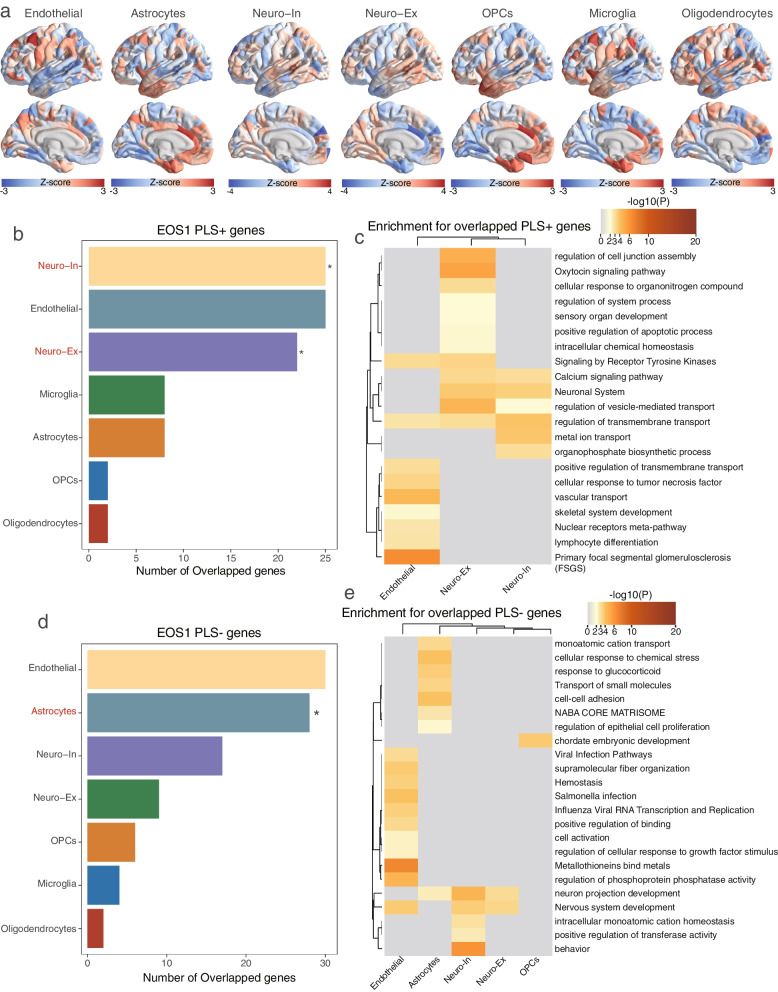
Cellular specific transcriptional signatures in accordance with MSN changes in EOS subtypes. **a**. The distribution of regional gene expression maps of seven brain cells using ssGSEA scores. **b**. The number of overlapping genes with PLS1 + weighted genes for each cell type, including inhibitory neurons (number = 25, adjusted *p*_perm_ = 0.021); excitatory neurons (number = 22, adjusted *p*_perm_ = 0.006); endothelial cells (number = 25, adjusted *p*_perm_ = 1); microglia (number = 8, adjusted *p*_perm_ = 0.825); astrocytes (number = 8, adjusted *p*_perm_ = 1); oligodendrocytes (number = 2, adjusted *p*_perm_ = 1); oligodendrocyte precursors (OPCs) (number = 2, adjusted *p*_perm_ = 1). **c.** Gene ontology and pathway terms enriched for PLS1 + weighted genes for different cell types. **d**. The number of overlapping genes with PLS1- weighted genes for each cell type, including inhibitory neurons (number = 17, adjusted *p*_perm_ = 0.9243); excitatory neurons (number = 9, adjusted *p*_perm_ = 1); endothelial cells (number = 30, adjusted *p*_perm_ = 0.9243); microglia (number = 4, adjusted *p*_perm_ = 1); astrocytes (number = 28, adjusted *p*_perm_ = 0.019); oligodendrocytes (number = 2, adjusted *p*_perm_ = 1); and oligodendrocyte precursors (OPCs) (number = 6, adjusted *p*_perm_ = 0.9243). **e**. Gene ontology and pathway terms enriched for PLS1- weighted genes for different cell types. All p values were obtained from permutation tests and adjusted with FDR correction

We next explored the transcriptional signatures at the cellular level along with the regional changes in MSN strength. We adopted an indirect method to match PLS1 weighted genes into seven canonical brain cell types, including endothelial cells, astrocytes, OPCs, microglia, oligodendrocytes, and excitatory and inhibitory neurons. The ssGSEA scores were used to summarize the gene expression of cells, displaying their distribution in different brain regions (Fig. 6a, Additional file 2: Table S9). In EOS1 patients, the PLS1 weighted gene list was significantly related to inhibitory neurons (number = 25, FDR-corrected adjusted *p*_perm_ = 0.021, FDR-corrected) and excitatory neurons (number = 22, FDR-corrected adjusted *p*_perm_ = 0.006) for PLS1 + genes and astrocytes (number = 28, FDR-corrected adjusted *p*_perm_ = 0.019) for PLS1- genes (Fig. 6b-d, Additional file 2: Table S10). Only a few overlapping genes were present in EOS2 cohorts between cells and the PLS1 weighted gene list without statistical significance. Based on cell-specific genes, the enrichment analysis revealed that MSN strength changes in EOS individuals were significantly enriched in BPs associated with signaling transport, as well as pathways of “calcium signaling”, “neuronal system” and “tyrosine kinases receptor for neuronal cells” (Fig. 6c). Astrocyte-specific genes were enriched in cellular response-related processes, including “cellular response to chemical stress”, “response to glucocorticoid”, “regulation of epithelial cell proliferation”, “cell–cell adhesion” and “Transport of small molecules” (Fig. 6e).

In addition, we evaluated the link between PLS weighted genes and developmental time windows across different brain regions in distinct EOS subtypes by developmental gene expression enrichment analysis, spanning from early fetal (EF) to young adulthood (YA) stages. The analysis revealed the PLS1 ± gene lists were predominantly expressed in brain regions from developmental terminal stages, particularly across specific areas including the cerebellum and thalamus for PLS1 + genes, and striatum, hippocampus, cortex, and amygdala for PLS1- genes in EOS1 patients (Additional file 1: Fig. S13 a-b). In contrast, no significant enrichment was present in any stages for PLS1 + genes in EOS2 patients, whereas PLS1- genes were primarily enriched in the early developmental stages in the cortex and amygdala areas (Additional file 1: Fig. S13 c-d). These findings identified specific cell types and distinct impressionable developmental stages, with distinct gene expression patterns according to the regional changes in MSN strength, providing maps of known specific cell types and developmental trajectories associated with EOS pathology.

## Discussion

This study is the first to conduct an integrated investigation of neuroanatomical subtypes of EOS patients by exploring the relationships between structural alterations and molecular mechanisms using a combined analysis of MSN macrostructural changes and specific transcriptional expression patterns. The study included 100 initially diagnosed and untreated EOS patients, along with 106 age- and gender-matched HC cohorts. We employed the novel machine learning approach (HYDRA) to identify two distinct MSN-based EOS subtypes, each exhibiting different abnormal MSN patterns compared with HC individuals. A combination of the AHBA and ISH datasets was used to identify 69 SCZ-related genes and further investigate the potential relationships between EOS-related regional changes in the MSN strength and corresponding transcriptomic patterns. Moreover, significant Spearman’s correlations were noted between PLS1 weighted genes of EOS-related MSN strength differences and DGEs from ASD, adult-SCZ, and IBD, and distinct functional enrichment of ontological terms and pathways in EOS subtypes was studied. Furthermore, the expression of PLS weighted genes was mapped to the distribution of astrocytes and neuronal cells, and BPs associated with these cells were identified using the Metascape software. These findings demonstrate the changes in the MSN strength across different EOS subtypes, bridging the gap between neuroimaging and transcriptional patterns and promoting an integrative understanding of EOS.

EOS1 displayed lower VIQ and FSIQ scores compared to EOS2, suggesting that cognitive characteristics distinguished the two specific EOS subtypes. In addition, similar subtypes were successfully identified in external adult-SCZ datasets with spatial positive correlation of MSN strength and shared abnormal regions, indicating the existence of disease subtypes. Moreover, the *t*-value distributions of the differences in the MSN strength between the two EOS subtypes and HCs were opposite. EOS1 displayed similarities to MDD [19] and adult-SCZ [20], with a typical distribution of differences in *t*-values for MSN strength. Unlike adult-SCZ patients, EOS1 exhibited more abnormally increased MSN strength in the frontal and parietal regions, with involvement of the occipital lobe and superior temporal gyrus. Furthermore, EOS1 exhibited more abnormally decreased MSN strength in the superior frontal and temporal regions, along with the involvement of the insula and precentral gyrus. However, EOS2 displayed the opposite atypical difference in the *t*-values of morphological similarity, with abnormally increased MSN strength only in the right paracentral gyrus and abnormally decreased MSN strength in the right superior frontal gyrus. EOS1 exhibited differences in MSN strength in multiple functional and von Economo atlas, whereas EOS2 displayed abnormalities only in MSN strength within motor networks. Previous studies have demonstrated that high/low MSN strength implies that the cellular structures of similar/differentiated networks could be more/less likely to form axonal connections to each other [41]. Changes in the brain develop rapidly from childhood to adolescence as myelination and synapse pruning [42, 43]. Consistent with these studies, our developmental trajectories analysis constructed a full landscape of MSN strength along with age development in different EOS subtypes. Abnormal morphological similarities in EOS1 suggested that EOS1 could be a typical subtype of EOS, whereas brain regions showed abnormal axonal connectivity before adulthood due to myelin and synaptic hypoplasia, with more severe scope and degree of abnormalities. Abnormal morphological similarities in EOS2 suggested EOS2 as an atypical subtype of EOS, demonstrating only a single abnormality of axonal connectivity. The abnormal MSN pattern of the two EOS subtypes indicated that, unlike the classification based on subjective clinical symptoms, the MSN-based classification of pure and drug-naïve EOS could enhance our understanding of the early neuropathological mechanism of SCZ.

The analysis of SCZ-related genes revealed significantly different transcriptional patterns in distinct EOS subtypes. In EOS1 patients, a substantial portion (46/69) of EOS-related genes were associated with regional changes in the MSN, whereas the EOS2 subtype exhibited only a few EOS-related genes (3/69), suggesting stronger connectivity between gene expression and structural abnormities in EOS1 than those in EOS2 patients. The discovered *CIT* gene encodes a citron rho-interacting kinase, which is known to regulate cytokinesis and central nervous system development [44]. A genetic mutation in the *CIT* (rs10744743) gene has been reported to be linked to risks for SCZ by interacting with the *DISC1* gene [45]. Similarly, the tachykinin precursor 1 (*TAC1)* gene has been implicated in the process of neuron excitation and behavioral responses by encoding four primary tachykinins, including substance-P, neurokinin-A, neuropeptide-K, and neuropeptide-γ [46, 47]. Interestingly, as shown in Fig. 3e, the *CIT* and *TAC1* genes exhibited contrary associations with corresponding changes in the MSN strength among different EOS subtypes, implying that expressional dysregulation of similar SCZ-related pathogenic genes were consistent with macrostructural abnormalities in brain regions with different EOS subtypes. In addition, the genetic polymorphism of the *CINR1* gene, widely expressed in the central nervous system, correlated with the pathogenesis of SCZ and antipsychotic response [48]. Furthermore, genome-wide association studies (GWAS) have identified variants of the *GRM7* gene as risk factors and responses to antipsychotic therapy in SCZ patients [49]. We demonstrated a significant spatial correlation between the expression of these genes and structural changes in the MSN strength. The positive/negative correlations indicated that PLS1 positively/negatively weighted genes were overexpressed in the macrostructures where the MSN strength was increased/decreased in EOS patients. These findings support a potential explanation that changes in transcriptional patterns may precede regional macrostructural abnormalities, and clinical disorder may be the latest during the development of SCZ.

Previous GWAS studies have reported shared genetic commonalities among major psychiatric disorders, particularly SCZ, BD, ASD, MDD, and obsessive–compulsive disorder [50, 51]. Moreover, a large-scale longitudinal study identified SCZ patients as high-risk groups for subsequent IBD development [52]. Similarly, Florian et al. demonstrated the presence of shared susceptibility genes (*NR5A2, SATB2*, and *PPP3CA*) between SCZ and IBD [53]. These studies suggest that common neural mechanisms or the brain-gut axis, facilitated by shared genetic commonalities could be linking multiple mental disorders. Furthermore, our MAGMA analysis identified enrichments of similar BPs at genetic levels in these neuropsychiatric disorders, particularly in energy metabolism and neural interaction signaling. Consistent with the potential genetic commonalities, our results revealed significant positive correlations between PLS1- weights and ASD-related and adult-SCZ-related DGE values in the EOS1 subtype and with IBD-related DGE values in the EOS2 subtype. In addition, these findings indicated that the EOS1 subtype exhibited characteristics of “classical SCZ” with similar psychic abnormalities, whereas the EOS2 subtype displayed the “non-classical SCZ” type with higher links to the genetic background of IBD.

Based on PLS1 weighted genes, the enrichment of BP terms and KEGG pathways provided insightful interpretations of transcriptional signatures concerning the regional changes in the MSN strength for different EOS subtypes. The PLS1 + weighted genes in EOS1 were enriched in similar ontological terms such as PLS1- weighted genes in EOS2 patients, including “regulation of cellular metabolic process”, “regulation of RNA metabolic process” and herpes simplex virus 1 (HSV-1) infection pathway. Multiple cellular metabolic processes have been associated with the dysfunction of the dorsal prefrontal cortex in SCZ [54], Similarly, HSV-1 infection is potentially associated with the pathogenesis of SCZ, as evident from infection and incubation in autonomic neurons and peripheral sensory neurons [55]. These results suggested that PLS1- weighted genes are prominently implicated in abnormal functions observed in EOS2 subtypes, and virtual infection could potentially explain the “nonclassical SCZ” status at the functional level. In addition to virtual infection, PLS1 + genes of EOS1 were enriched in calcium signaling and MAPK signaling pathways, which are implicated in the onset of SCZ by regulating neuronal excitability and neural development [56, 57]. However, PLS1- weighted genes in EOS1 were predominantly enriched in synaptic signaling-related functions, such as serotonergic synapse, phagosome, gap junction, neurotrophin signaling pathway. Synaptic signaling, responsible for synaptic stability and maturation, is intricately related to the mechanism of SCZ by regulating neuron connectivity, co-transmission, and activity [58]. This classical regulatory mechanism of SCZ can reasonably explain the “classical SCZ” status observed in EOS1 patients.

Cellular abnormalities have been known to play a vital role in the development of psychiatric disorders, including ASD, SCZ, BD, and MDD [59]. We identified inhibitory and excitatory neurons as the largest proportion among the seven cell types in PLS1 + weighted genes, whereas astrocytes constituted the majority among PLS1- weighted genes, concerning the changes in MSN strength in EOS1 patients. The distribution of the above cell types aligned with that of published studies using single-cell RNA sequencing (scRNA-seq) in SCZ [60], confirming transcriptional abnormities of these cells in the pathogenesis of SCZ. The imbalance between excitatory and inhibitory neurons is crucial for the pathogenesis of SCZ, especially GABAergic deficits, and excitation in glutamatergic systems [61]. Moreover, astrocytes are known to critically influence pivotal processes of neurodevelopment and homeostasis, leading to SCZ pathogenesis, induced by synaptogenesis, glutamatergic signaling, and myelination [62]. The functional enrichment analysis from previous studies has demonstrated that neurons were enriched in biological processes associated with signaling transport, as well as pathways of “calcium signaling”, “neuronal System” and “tyrosine kinases receptor for neuronal cells”. In contrast, astrocytes were enriched in the “regulation of epithelial cell proliferation”, “cell–cell adhesion” and “Transport of small molecules”, consistent with cellular functional annotation for SCZ. These findings provide us with a reliable pattern to investigate cellular transcriptional signatures in EOS patients.

Our study had several limitations. First, although machine learning methods were used to identify novel disease subtypes with optimal category numbers, the sample size of patients was relatively limited. Therefore, MSN macrostructural changes and specific transcriptional patterns in different EOS subtypes should be further estimated and validated through other congeneric studies. Second, we included a limited set of clinical parameters related to EOS symptoms. More comprehensive clinical signatures should be collected for correlation analyses with regional changes in the MSN strength from EOS subtypes, such as body mass index, hyperlipemia, and hyperglycemia. Finally, the transcriptional datasets, obtained from the AHBA database, only covered gene expression from two right hemisphere tissues, allowing us to include the datasets of only the left hemisphere in our analysis. Hence, there exists a lack of a corresponding connection between gene signatures and MSN macrostructural changes in the right hemisphere.

## Conclusions

In conclusion, we successfully identified two distinct EOS subtypes, each exhibiting distinct cognitive characteristics and MSN macrostructural changes compared to HC cohorts, linked to specific transcriptional patterns. The EOS1 subtype, termed as “classical SCZ” status, demonstrates consistent associations with SCZ-related genes, intricate relationships with DGEs of ASD and adult-SCZ, and functional signatures related to synapse signaling, along with abnormalities in neuron mechanisms. In contrast, the EOS2 subtype has the “non-classical SCZ” status, exhibiting significant associations with DGEs of IBD, and its pathogenesis could be interpreted by the HSV-1 infection pathway and abnormities of the astrocytes. Overall, this study provides a comprehensive understanding of neuroanatomical subtypes in EOS, shedding light on the intricate relationships between structural and molecular aspects of EOS. Furthermore, these findings provide valuable insights to advance our understanding of EOS and potentially guide future research and therapeutic approaches.

### Supplementary Information

**Additional file 1:** HYDRA method; **Figure. S1-S13.** **Figure. S1.** Cross-validated stability of EOS subtypes and adult-SCZ subtypes. **Figure. S2.** Comparison of WISC-CR between EOS subtypes and HC. **Figure. S3.** Distributions of MSN strength. **Figure. S4.** Regional changes in MSN strength between EOS1 and EOS2. **Figure. S5.** Yeo functional networks and von Economo atlas of subtyping EOS-control differences in the MSN strength (Bonferroni correction, *p* < 0.05). **Figure. S6.** Quadratic nonlinear model curve of the MSN strength development trajectory with age. **Figure. S7.** The spatial correlation analysis between case-control MSN maps of EOS subtypes and statistical maps of MSN strength and PANSS. **Figure. S8.** The spatial correlation analysis of case-control comparison of the MSN strength between EOS subtypes and adult-SCZ in Morgan et al. study. **Figure. S9.** Validation of disease subtype in adult-SCZ using datasets from Morgan et al. **Figure. S10.** Percent variance explanation of PLS in morphometric differences for EOS subtypes. **Figure. S11.** The supplements of correlation analysis between PLS1 weighted and transcriptional dysregulation of remaining mental disorders. **Figure. S12.** MAGMA enrichment for gene set enrichment of six psychiatric disorders using the GWAS summary datasets from FinnGen R9 database. **Figure. S13.** Transcriptional enrichment of developmental stages for EOS subtypes.

**Additional file 2:** **Table. S1-S10.** **Table S1.** The GWAS summary datasets of six psychiatric disorders from FinnGen R9 database. **Table. S2.** Regions showing abnormal MSN strength in EOS1 and EOS2 subtype. **Table. S3.** Pearson’s correlation analysis between PANSS score and abnormal MSN strength in EOS1 and EOS2 subtype. **Table. S4.** Results of PLS1 scores and case-control t-values in EOS1 and EOS2 subtype. **Table. S5.** Gene list of PLS1+ and PLS- genes in EOS1 and EOS2 subtype. **Table. S6.** The expression levels and spatial correlation of 69 overlapped SCZ-related genes. **Table. S7.** The DGEs of six mental disorders from published study. **Table. S8.** Results of GO and KEGG enrichment for PLS1+ and PLS1- genes in different EOS subtypes. **Table. S9.** Results of ssGSEA scores for different cells. **Table. S10.** Results of cellular overlapped genes with PLS1+ and PLS1- genes in different EOS subtypes.

## Data Availability

Data used in this study can be accessed on requirement from the corresponding authors.

## Abbreviations

EOS: Early-onset schizophrenia
MSN: Morphometric similarity network
HC: Healthy controls
HYDRA: Heterogeneity through discriminant analysis
PLS: Partial least squares regression
SCZ: Schizophrenia
MRI: Magnetic resonance imaging
AHBA: Allen Human Brain Atlas
ISH: In situ hybridization
DGEs: Differential gene expressions
DSM-IV: Diagnostic and Statistical Manual of Mental Disorders Structured Clinical Interview
PANSS: Fourth Edition, Positive and Negative Symptom Scale
FSPGR: Fast spoiled gradient-echo
TE: Time echo
TR: Time repetition
T1w: T1-weighted
TIV: Total intracranial volume
ARI: Adjusted Rand index
LRM: Linear regression model
FDR: False discovery rate
AAD: Alcohol abuse disorder
MDD: Major depressive disorder
BD: Bipolar disorder
ASD: Autism spectrum disorder
IBD: Inflammatory bowel disease
BP: Biological processes
GO: Gene ontology
KEGG: Kyoto Encyclopedia of Genes and Genomes
OPCs: Oligodendrocyte precursors
GWAS: Genome-wide association studies
HSV-1: Herpes simplex virus 1
