# Supplementary material for: Cortical structural changes of morphometric similarity network in early-onset schizophrenia correlate with specific transcriptional expression patterns
Source: BMC Med. 2023 Dec 5;21:479. doi: 10.1186/s12916-023-03201-1 (PMC10696871; doi:10.1186/s12916-023-03201-1)
Supplement: Supplementary file 1 — Additional file 1: HYDRA method; Figure. S1-S13. Figure. S1. Cross-validated stability of EOS subtypes and adult-SCZ subtypes. Figure. S2. Comparison of WISC-CR between EOS subtypes and HC. Figure. S3. Distributions of MSN strength. Figure. S4. Regional changes in MSN strength between EOS1 and EOS2. Figure. S5. Yeo functional networks and von Economo atlas of subtyping EOS-control differences in the MSN strength (Bonferroni correction, p < 0.05). Figure. S6. Quadratic nonlinear model curve of the MSN strength development trajectory with age. Figure. S7. The spatial correlation analysis between case-control MSN maps of EOS subtypes and statistical maps of MSN strength and PANSS. Figure. S8. The spatial correlation analysis of case-control comparison of the MSN strength between EOS subtypes and adult-SCZ in Morgan et al. study. Figure. S9. Validation of disease subtype in adult-SCZ using datasets from Morgan et al. Figure. S10. Percent variance explanation of PLS in morphometric differences for EOS subtypes. Figure. S11. The supplements of correlation analysis between PLS1 weighted and transcriptional dysregulation of remaining mental disorders. Figure. S12. MAGMA enrichment for gene set enrichment of six psychiatric disorders using the GWAS summary datasets from FinnGen R9 database. Figure. S13. Transcriptional enrichment of developmental stages for EOS subtypes. [file 12916_2023_3201_MOESM1_ESM.docx]

**HYDRA method**

The classification is executed by segregating healthy controls from patients through the creation of a convex polytope. This polytope is fashioned using linear maximum-margin classifiers. Then, subtyping is accomplished by clustering patients based on their association with various facets of this polytope, which are commonly denoted as hyperplanes. The HYDRA framework comprises several key stages: an initialization followed by iterations of assignment and polytope solutions, and the consensus of clustering results. Specifically, HYDRA initiates the clustering process by assigning patients to clusters. This initial assignment is achieved through the sampling of K unit-length hyperplanes, which are determined by considering the pairwise differences between patients and HC. The selection of the K unique hyperplanes is facilitated through the use of determinantal point processes (DPP), a sampling technique that effectively captures diverse disease-related directions. Subsequently, the sampled hyperplanes are employed to estimate the initial clustering assignments denoted as S-. Given the potential variation in estimated solutions based on the initial conditions, a multi-initialization strategy is implemented using DPP. Ultimately, the final clustering results are derived from a consensus of clustering solutions.

**Supplementary Figures**


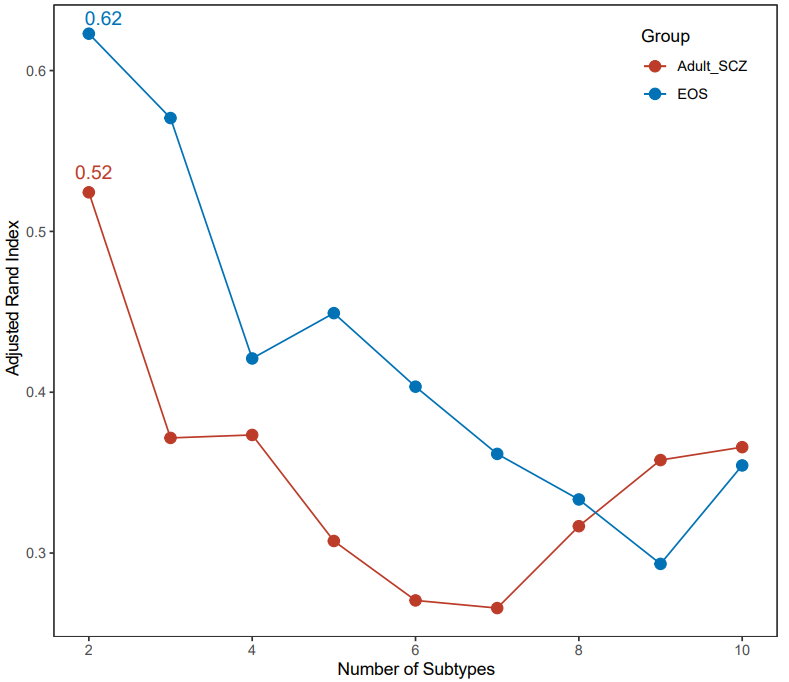


**Figure. S1.** **Cross-validated stability of EOS subtypes and adult-SCZ subtypes.** Adjusted Rand Index (ARI) vs. number of subtypes (K) indicates high reproducibility for K = 2.


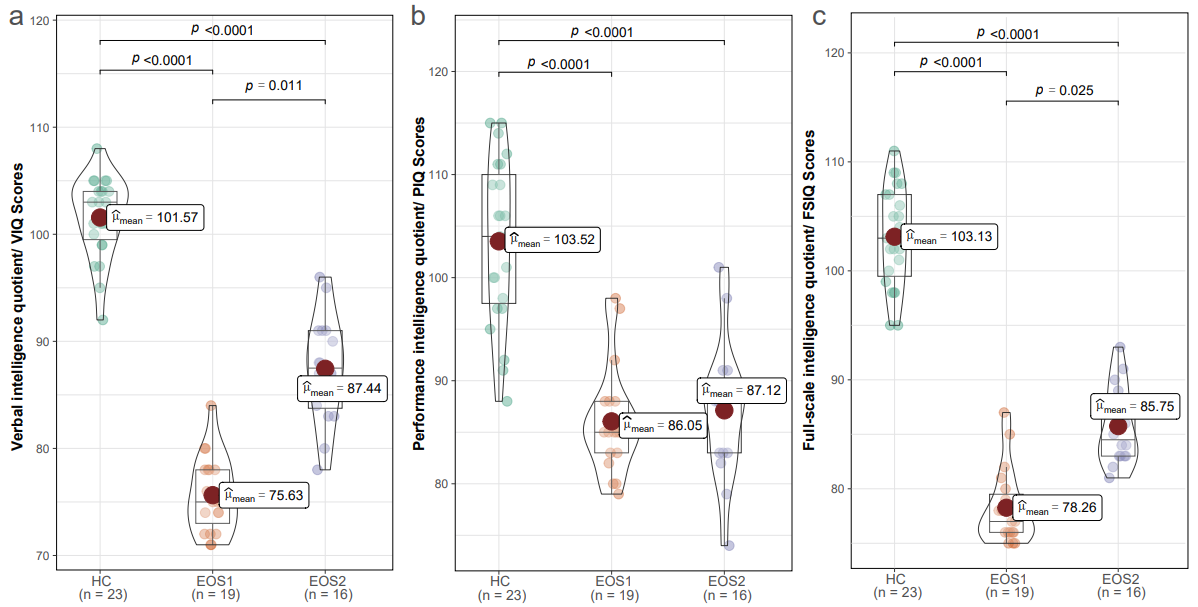


**Figure. S2. Comparison of Wechsler Intelligence Scale for children-Chinese Revised (WISC-CR) between EOS subtypes and HC. a-c.** The violin plot shows significant decrease of verbal (VIQ), performance (PIQ), and full-scale intelligence quotient (FSIQ) scores in both EOS subtypes compared to HC. Compared to EOS2, EOS1 exhibited lower VIQ and FSIQ scores and no significant difference in PIQ scores.


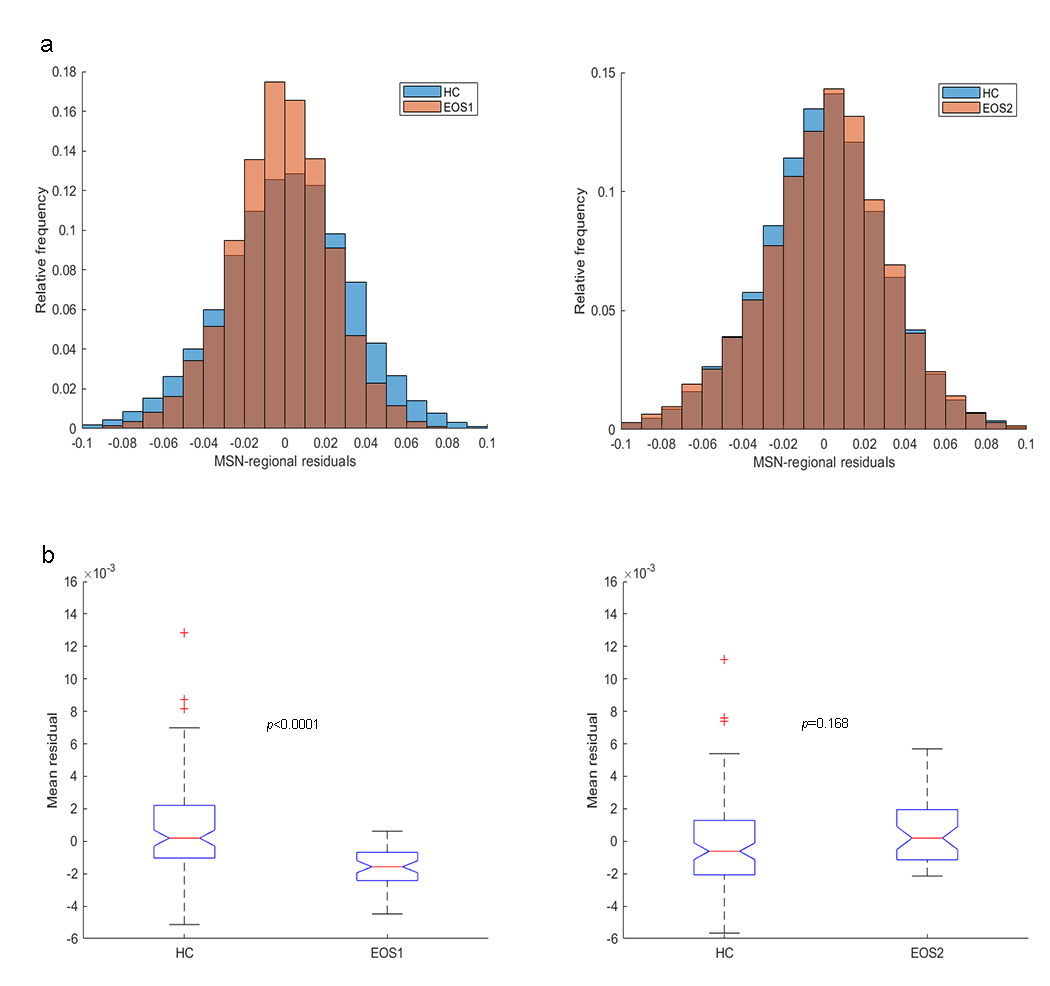


**Figure. S3. Distributions of MSN strength.** **a.** Distributions of MSN strength; **b.** Box plots for global mean MS. The global MSN of EOS1 exhibited a significant difference (*t* = -6.047, *p* < 0.001) compared to HC, while the global MSN of EOS2 showed no significant difference (*t* = 1.386, *p* = 0.168) compared to HC.


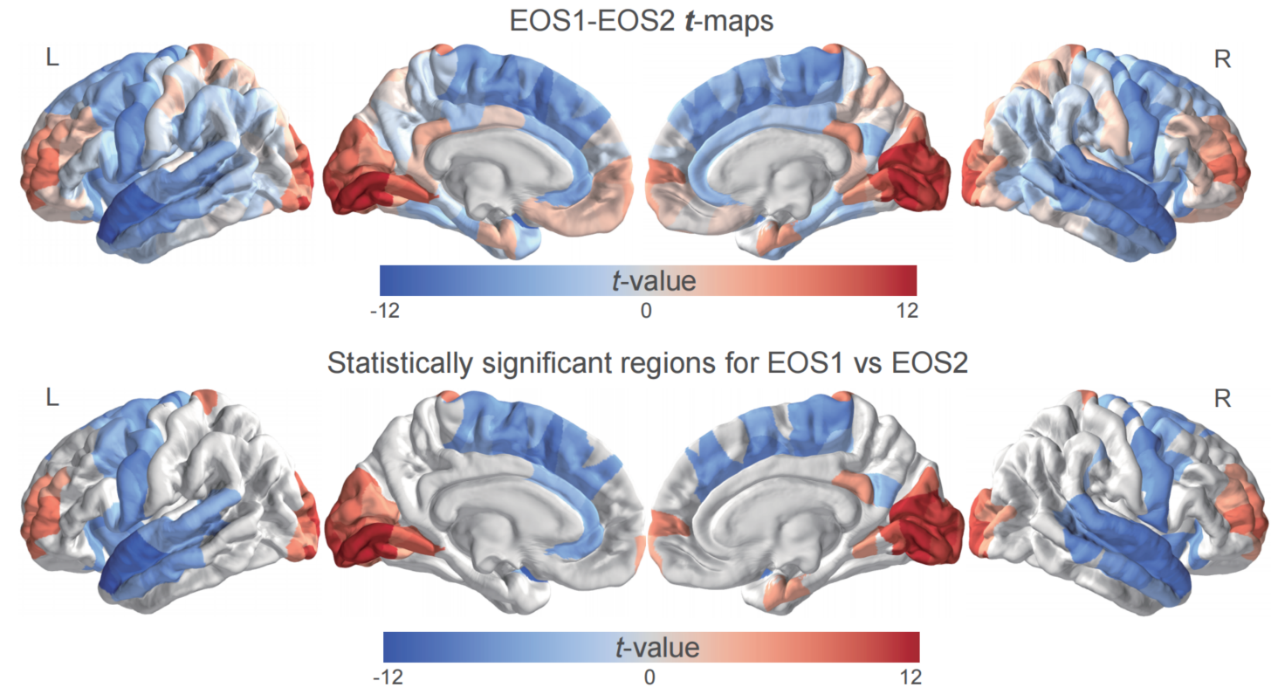


**Figure. S4.** **Regional changes in MSN strength between EOS1 and EOS2.** Compared to EOS2, EOS1 showed decreased MSN strength in the superior frontal gyrus, middle frontal gyrus, insula, and anterior central gyrus, and increased MSN strength in the lateral occipital cortex, lingual gyrus, and cuneus.

**
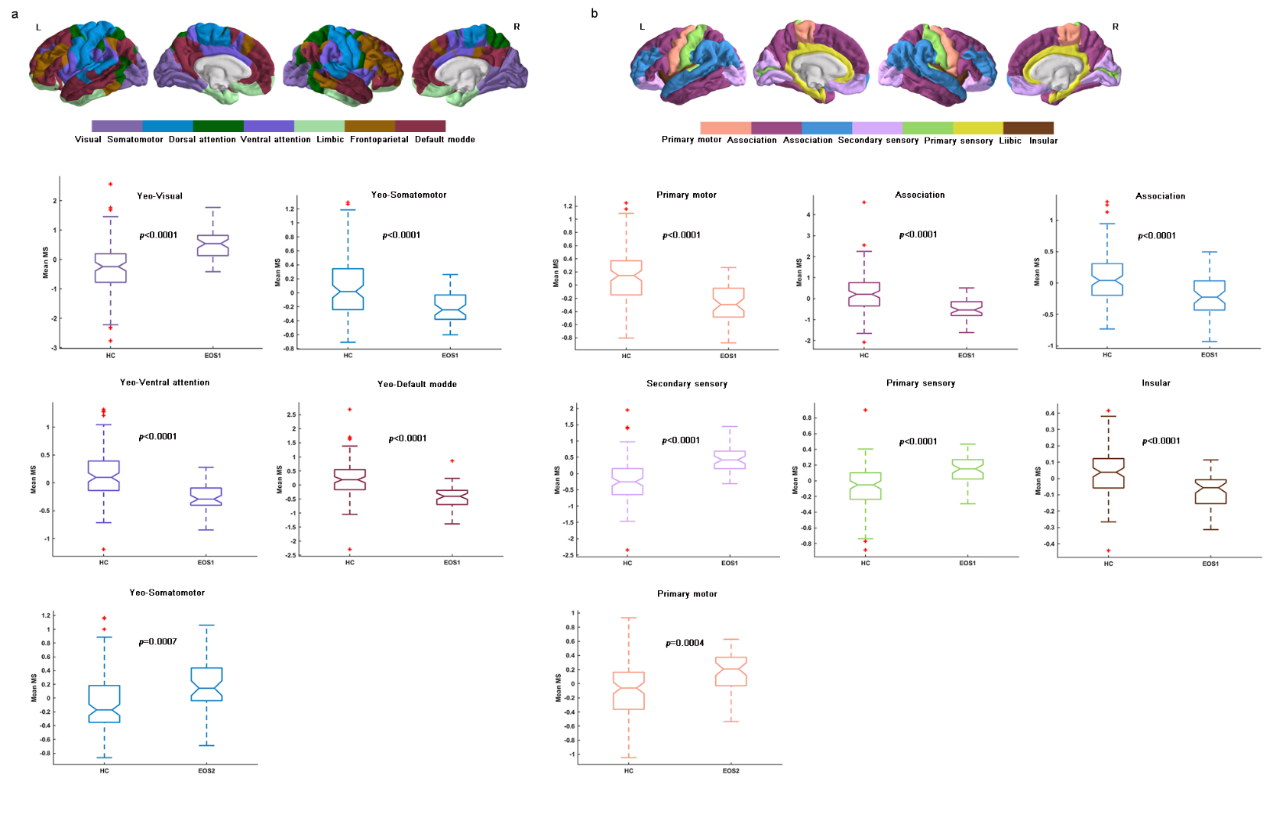
****Figure. S5.** **Yeo functional networks and von Economo atlas of subtyping EOS-control differences in the MSN strength (Bonferroni correction, *p* < 0.05).** **a.** The Yeo 7 functional networks; **b.** The von Economo **atlas**.


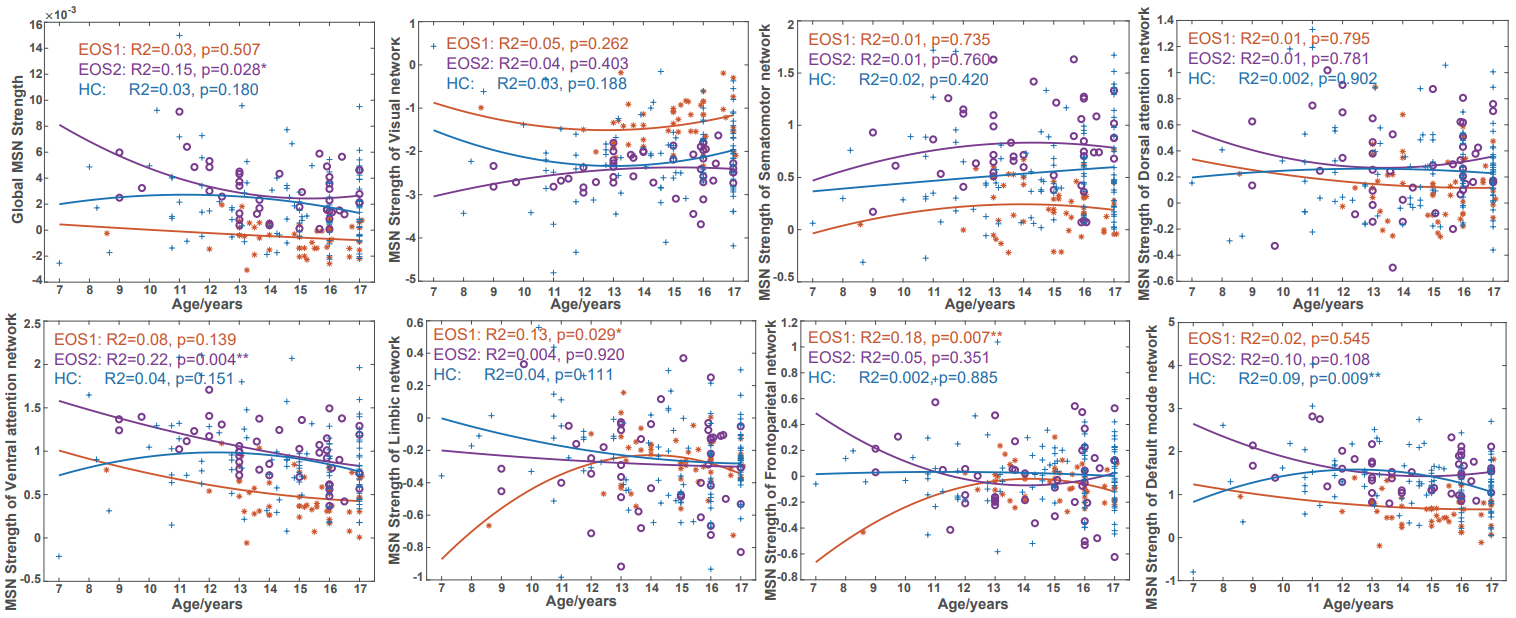


**Figure. S6.** Quadratic nonlinear model curve of the MSN strength development trajectory with age. **p* <0.05; ***p* <0.01.

**
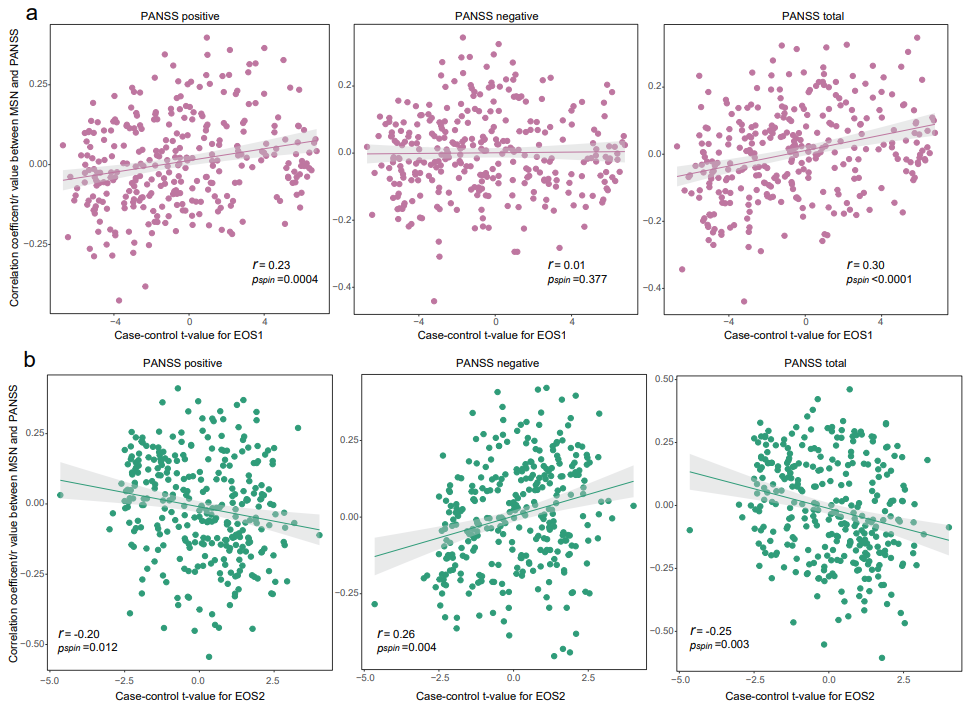
**

**Figure. S7. The** **spatial correlation analysis between case-control MSN maps of EOS subtypes and statistical maps of MSN strength and PANSS. a.** In EOS1 patients, the case-control MSN maps exhibited significant spatial positive-correlation with statistical maps in PANSS positive and total scores but not with negative scores. **b.** In EOS2 patients, the case-control MSN maps exhibited significant spatial negative-correlation with PANSS positive and total scores, while positive-correlation with PANSS negative scores.

**
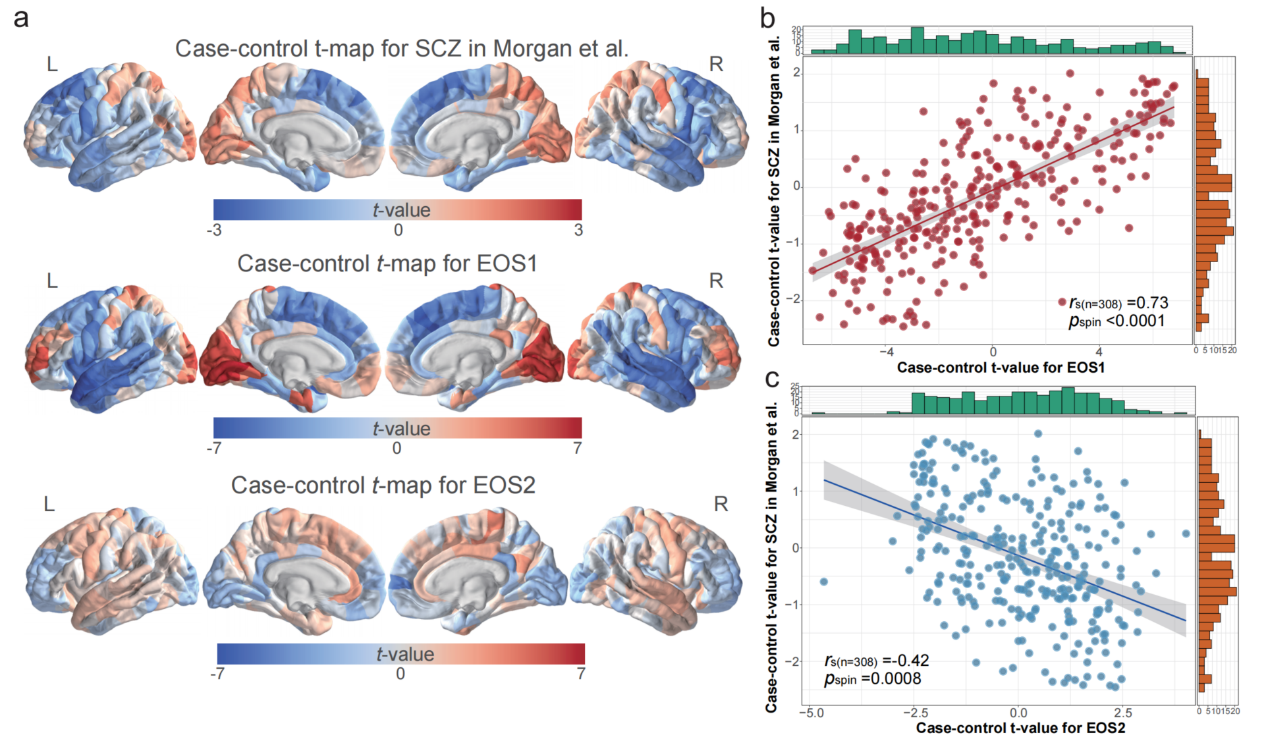
**

**Figure. S8.** **The spatial correlation analysis of case-control comparison of the MSN strength between EOS subtypes and adult-SCZ in Morgan et al. study. a.** Case-control *t*-maps of the MSN strength for adult-SCZ in Morgan et al. study, EOS1 and EOS2. **b-c.** The scatter plots show that EOS1 exhibits significant spatial positive-correlation (b) while EOS2 displays significant spatial negative-correlation (c) with case-control *t*-map of adult-SCZ in Morgan et al.

**
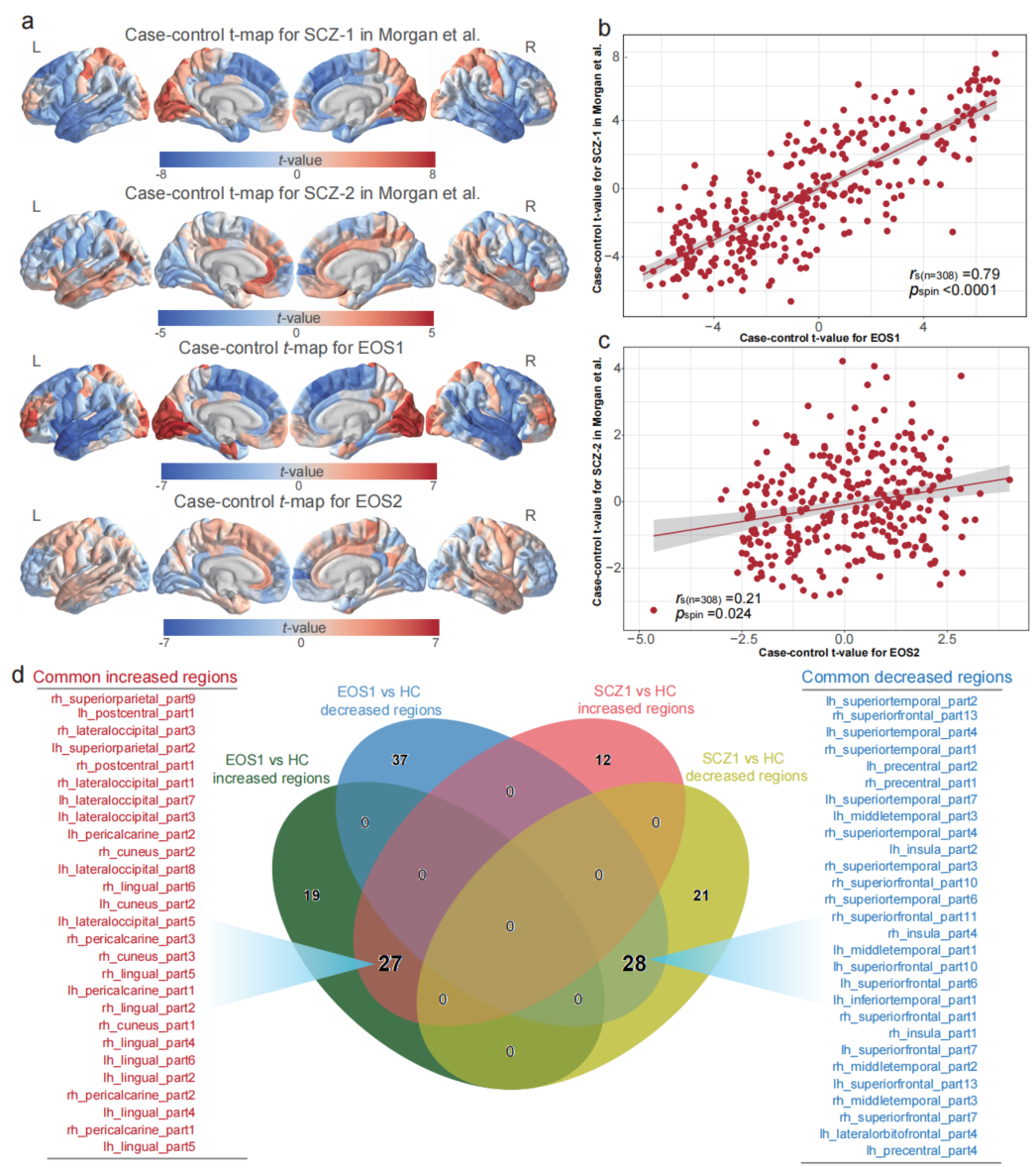
**

**Figure. S9. Validation of disease subtype in adult-SCZ using datasets from Morgan et al. a.** Case-control *t*-map of the MSN strength for adult-SCZ and EOS subtypes. **b-c.** The spatial correlation analysis shows significant spatial positive-correlations of case-control *t*-map in both EOS1/2 and SCZ1/2 subtypes. **d.** Veen plot displays common abnormal regions between type-I disease and HC cohorts, including 27 increased and 28 decreased regions.

**
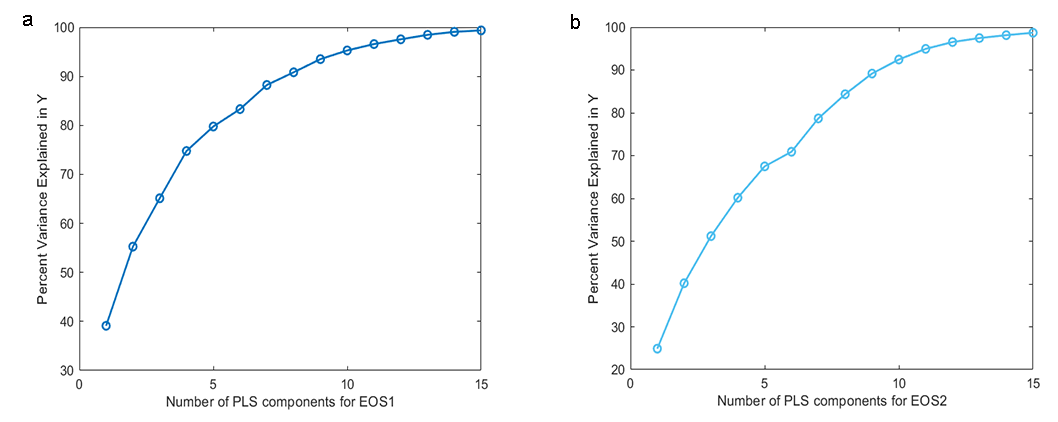
**

**Figure. S10.** Percent variance explanation of partial least squares (PLS) in morphometric differences for EOS1 (PLS1 = 0.39) and EOS2 (PLS1 = 0.25).


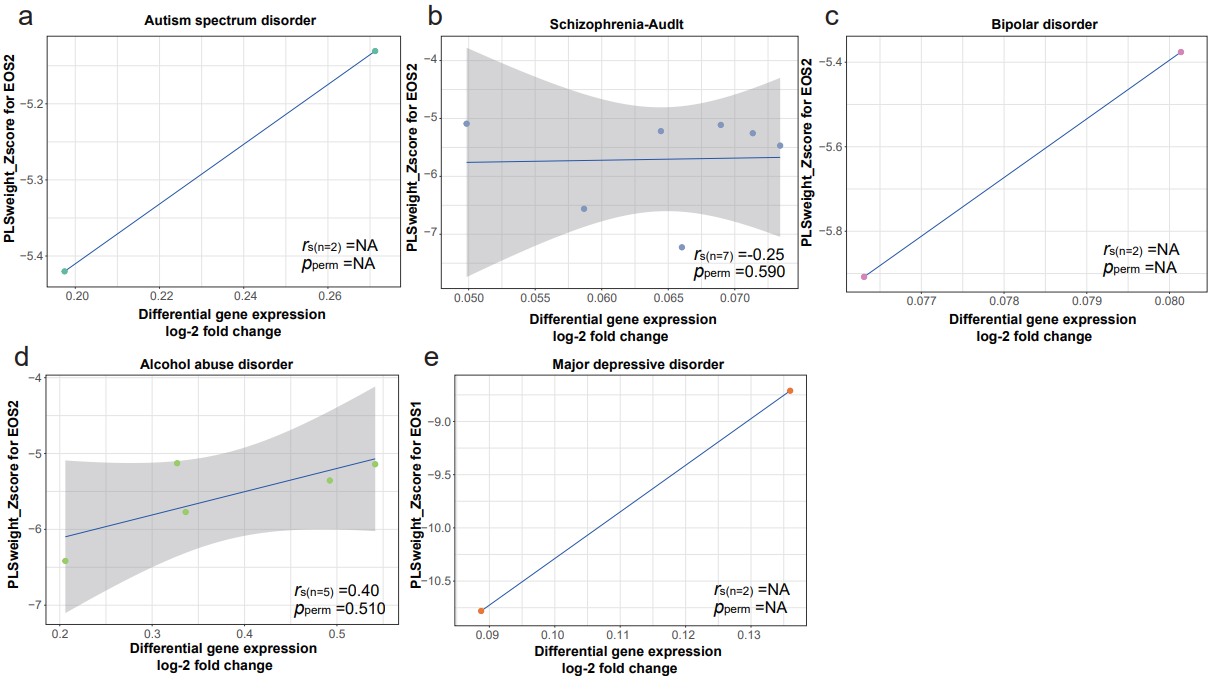


**Figure. S11. The supplements of correlation analysis between PLS1 weighted and transcriptional dysregulation of remaining mental disorders.** Only few common genes were identified and there was no significant statistical difference using the Permutation test.


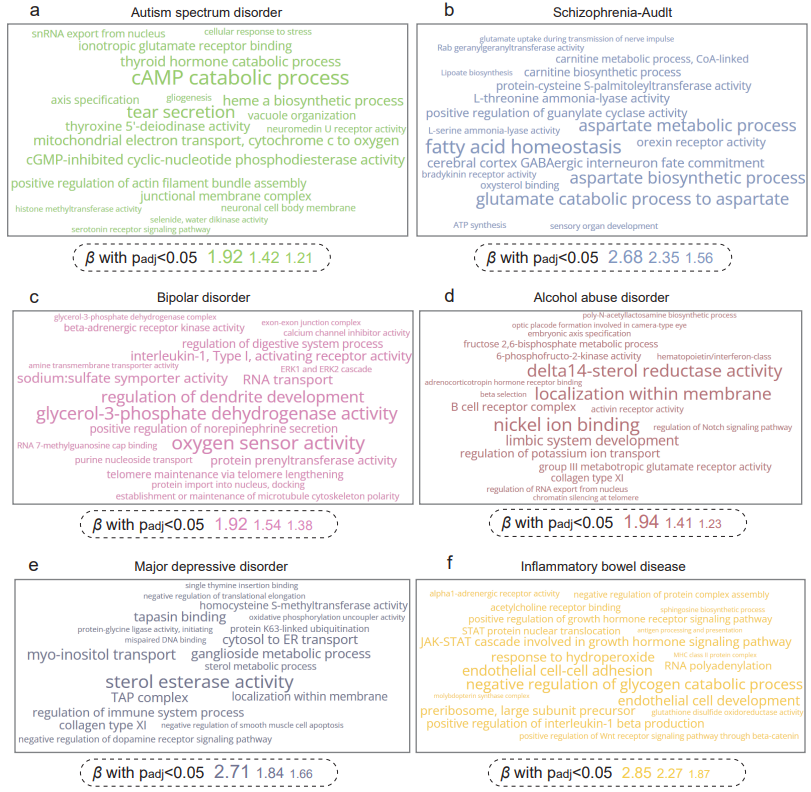


**Figure. S12. MAGMA enrichment for gene set enrichment of six psychiatric disorders using the GWAS summary datasets from FinnGen R9 database. a-f.** The cloud-word diagram displays the top20 enrichments of biological process with adjusted p<0.05. The size of words represents the β values of corresponding process.


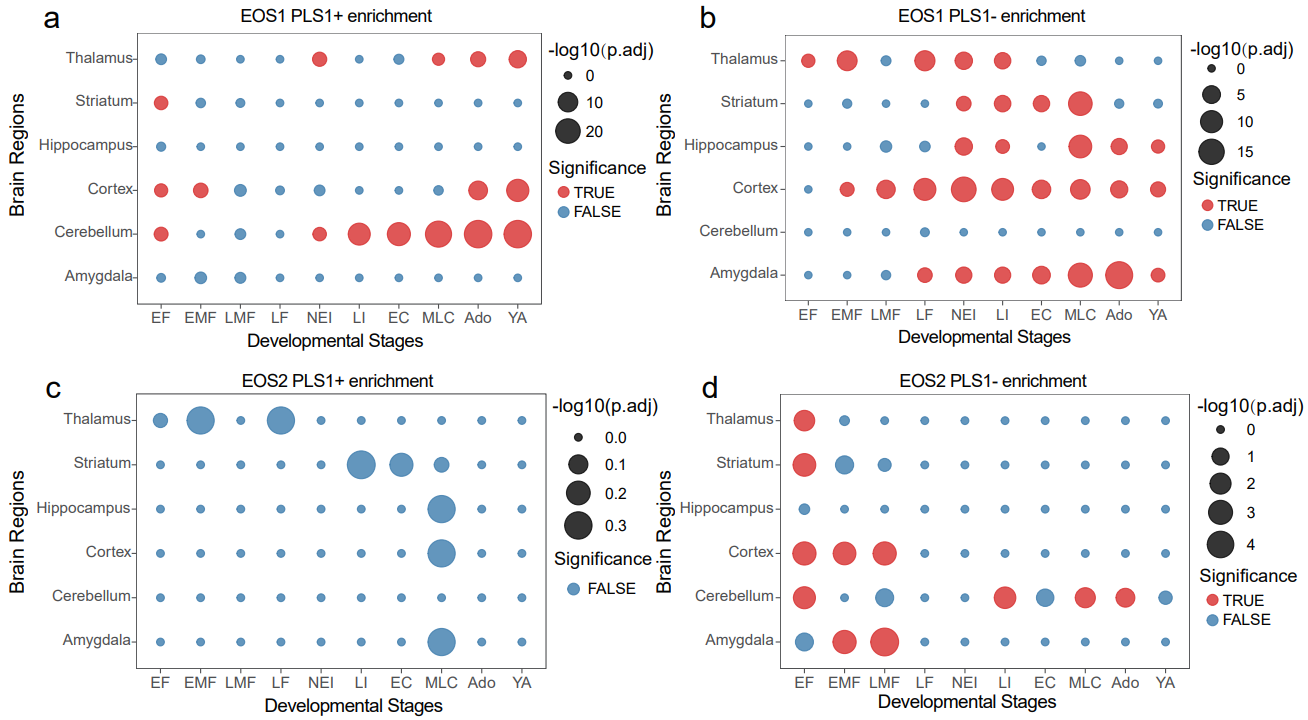


**Figure. S13. Transcriptional enrichment of developmental stages for EOS subtypes. a-b.** Developmental gene expression enrichment analysis showing that the PLS1 weighted genes exhibited predominant expression in the brain regions during the terminal stages (from late fetal (LF) to young adulthood (YA)) in EOS1 patients (a-b). **c-d.** In EOS2 subtypes, there was no significant enrichment in any stages for PLS1+ genes while PLS1- genes were primarily enriched in early developmental stages (from early fetal (EF) to late mid fetal (LMF)) (c-d). The size of nodes represents the adjusted p values.
